# Quality of Life After Percutaneous Coronary Intervention Versus Coronary Artery Bypass Grafting

**DOI:** 10.1161/JAHA.123.030069

**Published:** 2023-11-20

**Authors:** Arnaldo Dimagli, Cristiano Spadaccio, Annie Myers, Michelle Demetres, Tessa Rademaker‐Havinga, Gregg W. Stone, John A. Spertus, Bjorn Redfors, Stephen Fremes, Mario Gaudino, Ruth Masterson Creber

**Affiliations:** ^1^ Bristol Heart Institute, University of Bristol Bristol United Kingdom; ^2^ Department of Cardiothoracic Surgery Weill Cornell Medical College New York NY; ^3^ Mayo Clinic Department of Cardiovascular Surgery Rochester MN; ^4^ Columbia University Irving Medical Center New York NY; ^5^ Cardialysis Rotterdam The Netherlands; ^6^ Zena and Michael A. Wiener Cardiovascular Institute, Icahn School of Medicine at Mount Sinai New York NY; ^7^ Saint Luke’s Mid America Heart Institute, University of Missouri Kansas City MO; ^8^ Sahlgrenska University Hospital Gothenburg Sweden; ^9^ Sunnybrook Health Sciences Center University of Toronto Toronto Canada; ^10^ Columbia University School of Nursing Columbia University Irving Medical Center New York NY

**Keywords:** coronary artery bypass grafting, coronary artery disease, percutaneous coronary intervention, Cardiovascular Surgery, Meta Analysis, Quality and Outcomes

## Abstract

**Background:**

Differences in quality of life (QoL) after coronary artery bypass grafting (CABG) compared with percutaneous coronary intervention (PCI) are not well characterized. We aimed to compare the short‐ and long‐term effects of CABG versus PCI on QoL.

**Methods and Results:**

We performed a systematic review and meta‐analysis of randomized controlled trials comparing CABG versus PCI using the Seattle Angina Questionnaire (SAQ)‐Angina Frequency, SAQ‐QoL, SAQ‐Physical Limitations, EuroQoL‐5D, and Short‐Form Questionnaire. We calculated mean changes within each group from baseline to 1, 6, 12, and 36 to 60 months (latest follow‐up) and the weighted mean differences between groups using inverse‐variance methods. A total of 10 760 patients were enrolled in 5 trials. From baseline to 12 months and 36 to 60 months, the mean change in SAQ‐Angina Frequency was >22 points (95% CI, 21.0–25.6) after both PCI and CABG. The mean difference in SAQ‐Angina Frequency was similar between procedures at 1 month and at 36 to 60 months but favored CABG at 12 months (1.97 [95% CI, 0.68–3.26]). SAQ‐QoL favored PCI at 1 month (−2.92 [95% CI, −4.66 to −1.18]) and CABG at 6 (2.50 [95% CI, 1.02–3.97]), 12 (3.30 [95% CI, 1.78–4.82]), and 36 to 60 months (3.17 [95% CI, 0.54 5.80). SAQ‐Physical Limitations (−12.61 [95% CI, −16.16 to −9.06]) and EuroQoL‐5D (−0.07 [95% CI, −0.08 to −0.07) favored PCI at 1 month. Short‐Form Questionnaire‐Physical Component favored CABG at 12 months (1.18 [95% CI, 0.46–1.90]).

**Conclusions:**

Both PCI and CABG improved long‐term disease‐specific and generic QoL.

**Nonstandard Abbreviations and Acronyms**
AF: angina frequency
EQ‐5D: EuroQoL‐5D
MCS: mental component score
PCS: physical component score
SAQ: Seattle Angina Questionnaire
SF‐12/36: Short Form Health Survey‐12/36

**Clinical Perspective**

**What Is New?**

- This is the first meta‐analysis comparing short and longer‐term quality of life after percutaneous coronary intervention and coronary artery bypass grafting.
- In the first few months after both interventions, improvements in quality of life favored percutaneous coronary intervention, then coronary artery bypass grafting by 1 year, and the outcomes were similar between percutaneous coronary intervention and coronary artery bypass grafting by 3 to 5 years.

**What Are the Clinical Implications?**

- Quality of life is an important component of patient‐centered care; measuring both generic and disease‐specific quality of life should be part of the shared decision‐making before cardiac interventions and during the recovery phase.

Coronary artery disease (CAD) is the leading cause of global mortality and morbidity.^1^ Revascularization strategies to address CAD include percutaneous coronary intervention (PCI) and coronary artery bypass grafting (CABG), with PCI generally favored in noncomplex disease and CABG favored when extensive CAD or diabetes is present. There have been several clinical trials comparing PCI and CABG in chronic CAD, and several meta‐analyses have been conducted focusing on survival and event‐free survival. Less is known about differences in quality of life (QoL) between the interventions across these trials.

Patient‐reported outcomes (PROs) provide a holistic picture of where patients are on their disease trajectory. PROs can be used for routine management, to monitor recovery after major clinical interventions,^2^ and for shared decision‐making between patients and health care professionals. PROs are being increasingly emphasized as relevant end points in clinical trials because a primary treatment goal is to optimize patients' overall health status, symptom control, function, and QoL.^3^, ^4^ The latter has recently been endorsed as a primary recommendation by updated revascularization guidelines, but how to inform patients on the relative health status benefits of PCI or CABG has not been described.^3^, ^5^

The objective of this study was thus to evaluate the differences in generic and disease‐specific QoL in patients with CAD treated by CABG or PCI. This meta‐analysis builds on our previous meta‐analytic work describing changes in QoL and symptoms of angina frequency up to 1 year after CABG.^6^ While other meta‐analyses of PCI and CABG QoL outcomes relied on observational studies, which are subject to unmeasured confounding,^7^, ^8^ we limited the present systematic review and meta‐analysis to randomized clinical trials comparing QoL between CABG and PCI to eliminate potential selection biases in patient treatment.

## METHODS

The data underlying this article will be shared upon reasonable request to the corresponding author.

This study was performed following the Preferred Reporting Items for Systematic Reviews and Meta‐Analyses (PRISMA) statement.^9^ In adherence with these guidelines, a protocol was registered in PROSPERO, an international prospective register of systematic reviews (CRD42020221338). This meta‐analysis was deemed exempt by the Weill Cornell Medicine institutional review board. Informed consent was not required because this was a study‐level meta‐analysis, and no human subjects were involved.

### Search Strategy

A medical librarian (M.D.) performed comprehensive searches to identify studies that evaluated the impact of CABG surgery on QoL. Searches were run on May 6, 2021, in the following databases: Ovid MEDLINE (ALL 1946 to present); Ovid EMBASE (1974 to present); Cochrane Library (Wiley); PsycINFO (EBSCO); and CINAHL (EBSCO). Search terms included all subject headings and associated keywords for the concepts of PCI, CABG, and QoL and were then limited to randomized controlled trials using a validated filter. The full search strategy for Ovid MEDLINE is available (Table S1).

### Selection Criteria

After results were de‐duplicated, at least 2 independent reviewers (A.D., C.S., A.M., R.M.C.) screened titles and abstracts. Discrepancies were resolved by consensus with a third reviewer. Titles and abstracts were reviewed against a priori inclusion/exclusion criteria. Articles considered for inclusion were randomized controlled trials (RCTs) that measured disease‐specific QoL and symptoms using Seattle Angina Questionnaire (SAQ) and generic QoL using Short Form Health Survey‐12/36 and EuroQoL‐5D (EQ‐5D) at baseline and follow‐up, published in English from 2000 to 2021. There were no exclusion criteria based on coronary disease (multivessel versus left main disease) or clinical presentation (acute versus stable coronary disease). Full text articles were reviewed in a second round of eligibility screening. Reference lists and articles citing the included studies were extracted from Scopus (Elsevier) (Figure S1).

### Data Extraction

Four reviewers (A.D., A.M., C.S., R.M.C.) abstracted quantitative data from selected studies, and each extraction was made independently by 2 separate reviewers with inconsistencies resolved by consensus. Extracted items included study demographics (sample size, publication year, country, study period, number of centers), baseline patient characteristics, comorbidities (age, sex, race, diabetes, hypertension, chronic obstructive pulmonary disease, peripheral vascular disease, history of previous myocardial infarction), and QoL values at baseline, 1, 6, 12 months, and at latest follow‐up (ranging from 36–60 months in various studies) after PCI or surgery.

The QoL assessments extracted were the disease‐specific SAQ and 2 generic QoL measures, the EQ‐5D and Short Form Health Survey‐12/36. The Short Form Health Survey‐12/36 and EQ‐5D emphasize generic (overall) QoL, including physical and mental health symptoms and social roles, while the SAQ is focused on disease‐specific symptoms, namely, angina, the most common symptom‐related benefit of revascularization.

The SAQ is a validated 19‐item questionnaire that measures 5 domains related to CAD (angina frequency [AF], physical limitations [PL], QoL, angina stability, and treatment satisfaction), with scores ranging from 0 to 100 (higher scores indicate less symptom burden).^10^ The 3 most relevant SAQ domains were included in this study (AF, QoL, and PL). The EQ‐5D is a 5‐item instrument to assess general health status in the following 5 dimensions: mobility, self‐care, usual activity, pain or discomfort, and anxiety or depression.^11^

EQ‐5D scores range from 0 to 100 and can be converted to an index score ranging from 0 (worst health status) to 1 (best possible health status).^12^ The summary score of the EQ‐5D was used in the analyses.

The SF‐12 and SF‐36 measure 8 dimensions of health: physical functioning, role limitations due to physical problems, bodily pain, vitality, general health perception, social function, role limitations due to emotional problems, and mental health.^13^, ^14^ Scores for each domain range from 0 to 100, with higher scores indicating better health status. The SF‐12 and SF‐36 have 2 summary measures: a physical component score (PCS) and mental component score (MCS), with norm‐based methods for age and sex that standardize the score to a mean of 50 and SD of 10. If data were not available in the published articles, we reached out to authors for aggregated, unpublished data on the outcomes. Risk of bias was assessed through the Cochrane risk‐of‐bias tool for RCTs.^15^

### Outcomes

The primary study outcome was the weighted mean gain of SAQ‐AF from the pre‐intervention baseline score to 12 months after intervention. Secondary outcomes were the weighted mean gains of SAQ‐AF at 1, 6, and 36 to 60 months and weighted mean gains of SAQ‐QoL, SAQ‐PL, EQ‐5D, Short Form Health Survey‐12/36 PCS, and MCS from baseline to 1, 6, 12, and 36 to 60 months. We combined the 36‐ and 60‐month time points because they represented the last available follow‐up time point for the included trials and could not be analyzed singularly due to the paucity of studies at each of those time points.

### Statistical Analysis

Continuous variables were extracted from the 5 RCTs as mean ± SD or median (interquartile range). Median and interquartile range were converted to mean ± SD if the mean values were not available and the median interquartile range was available using the formulas from the Cochrane Handbook.^16^ Categorical variables were reported as frequencies (percentages).

For each trial, we first calculated the difference in scores from baseline to 1, 6, 12, and 36 to 60 months within the CABG and PCI groups (within‐group mean changes). The mean change was calculated as the difference between the mean score at the specified time point (1, 6, 12, or 36–60 months) and the mean score at baseline:

$${\text{Mean}}_{\text{change}}={\text{Mean}}_{\text{time point}}-{\text{Mean}}_{\text{baseline}}$$

The SD associated with the mean change was calculated using the formula provided by Cochrane^16^ where *r* represents the correlation coefficient and was given a conservative value of 0.5 in the analysis when not provided by the single studies.

$${\mathrm{SD}}_{\text{change}}=\sqrt{{\mathrm{SD}}_{\text{baseline}}^{2}+{\mathrm{SD}}_{\text{time point}}^{2}-\left(2\times r\times {\mathrm{SD}}_{\text{baseline}}\times {\mathrm{SD}}_{\text{time point}}\right)}$$

We then calculated the difference of within‐group mean changes between CABG and PCI (between‐group mean differences). These differences were weighted and pooled using an inverse‐variance method. Thus, each study was given a weight equal to the inverse of the variance of the effect estimate so that studies with smaller SE were given more weight than studies with larger SE, thereby reducing the uncertainty around the pooled effect estimates.^17^ The pooled estimate was called “mean gain.” Results are presented as mean gains with a 95% CI.

Statistical heterogeneity was assessed using *I* ^2^ and χ^2^ tests. We considered significant statistical heterogeneity if *I* ^2^ >80% and χ^2^ *P* value was <0.05. We conducted both a random‐effect and a fixed‐effect model for the overall summary of the treatment effect across studies. The random‐effect model was considered the primary analysis for the study, in part to account for potential clinical heterogeneity, reduce the risk of overstating uncertainty, and support the generalizability of the results beyond the included studies.^18^, ^19^

Random effects meta‐regression was performed to assess the effects of covariates (sex, age, diabetes, baseline QoL score, and year of publication) as independent variables on changes in QoL between groups. There were insufficient data to perform subgroup analyses by type of CABG procedure. As a sensitivity analysis, we performed a leave‐1‐out approach to assess how excluding 1 study at a time impacts the overall pooled estimates. When a study did not report the sample size at the follow‐up time points, we assumed it was equal to the baseline sample size.

Hypothesis testing was set at the 2‐tailed 0.05 level. All *P* values are 2‐sided, and *P* values <0.05 were considered to indicate statistical significance. There was no prespecified plan to adjust for multiple comparisons, given the high correlation across the different PROs. Given the significance threshold, the expected rate of significant results due to chance is 5%. Analyses and data modeling were performed with R‐project (version 3.3.3, R project for Statistical Computing), using the following packages: ‘meta,’ ‘dmetar,’ ‘robvis.’

## RESULTS

### Study and Participant Characteristics

A total of 3313 titles and abstracts were screened, 140 full text articles were reviewed, and 8 were included in this meta‐analysis representing 5 clinical trials. A total of 5415 patients had a PCI, and 5345 patients had a CABG in 5 RCTs from >14 countries in Europe and North America (Table 1).^20^, ^21^, ^22^, ^23^, ^24^, ^25^, ^26^ One study had moderate and 7 had low risk of bias (Figure S2, ^20^, ^21^, ^22^, ^23^, ^24^, ^25^, ^26^, ^27^).

**Table 1:** Study Characteristics

Trial	First author (y)	Country	Study period	Intervention/comparison group	Outcome assessments	Follow‐up time points (mo)
FREEDOM	Abdallah (2013)^20^	Worldwide	2005–2010	CABG vs PCI	SAQ	B, 1, 6, 12
	Magnuson (2013)^21^				EQ‐5D	
SYNTAX	Cohen (2011)^22^	Austria, Belgium, Denmark, Finland, France, Germany, Hungary, Italy, Netherlands, Poland, Spain, Sweden, UK, USA	2005–2007	CABG vs PCI	SAQ, SF‐36	B, 1, 6, 12
	Cohen (2014)^23^				EQ‐5D	B, 1, 6, 12, 36, 60
	Abdallah (2017)^27^				SAQ, SF‐36	B, 1, 6, 60
EXCEL	Baron (2017)^24^	Worldwide	2010–2014	CABG vs PCI	SAQ, SF‐36, PHQ‐8	B, 1, 12, 36
SoS	Zhang (2003)^25^	Europe, Canada	1996–1999	CABG vs PCI	SAQ	B, 6, 12
ARTS	Serruys (2001)^26^	Netherlands, Brazil, UK	1997–1998	CABG vs PCI	EQ‐5D, SF‐36	B, 1, 6, 12, 36

ARTS indicates Arterial Revascularization Therapies Study; B, baseline; CABG, coronary artery bypass grafting; EQ‐5D, EuroQoL‐5D; PCI, percutaneous coronary intervention; EXCEL, Evaluation of XIENCE versus Coronary Artery Bypass Surgery for Effectiveness of Left Main Revascularization; FREEDOM, Future Revascularization Evaluation in Patients with Diabetes Mellitus: Optimal Management of Multivessel Disease; PCI, percutaneous coronary intervention; PHQ‐8, Patient Health Questionnaire; SAQ, Seattle Angina Questionnaire; SF‐36, 36‐item Short‐Form Health Survey; SoS, Stent or Surgery Trial; and SYNTAX, Synergy between PCI with Taxus and Cardiac Surgery.

The sample sizes in the individual studies ranged from 235 to 945 participants in the PCI and from 231 to 935 participants in the CABG groups, respectively. Baseline patient characteristics are included in Table 2. The mean age was 64 years, and less than one‐third were female. The proportion of patients with diabetes varied from 16% to 100% across studies, and 18% to 44% had a previous myocardial infarction. There was a wide range in statistical heterogeneity across the studies (*I* ^2^: 0%–99%).

**Table 2:** Baseline Characteristics of Patients Included in the Studies

First author (y)	PCI									CABG								
	N	Age (y)	Sex (%M)	Race (%)	Diabetes (%)	Hypertension (%)	COPD (%)	MI (%)	PVD (%)	N	Age (years)	Sex (%M)	Race (%)	Diabetes (%)	Hypertension (%)	COPD (%)	MI (%)	PVD (%)
Abdallah (2013)^20^	945	63.2	73.2	White: 51.9	100		3.4	26.3	10.1	935	63	69.8	White: 50.8	100		5.5	25.1	10.5
Abdallah (2017)^27^	883	65.2	76.2		9.6			32.1		848	64.8	79.4		10.1			34.6	
Baron (2017)^24^	892	66	76.8		29	77.2		18.4		896	65.8	78.2		28.6	76.3		16.9	
Cohen (2011)^22^	903	65.2	76.4	White: 97.0	28.2			31.9		897	65	78.9	White: 95.5	28.5			33.8	
Cohen (2014)^23^	896	65.3	76.6		28.3		7.9	32.1	9.2	870	64.9	79.4		27.6		9.2	33.3	10.5
Magnuson (2013)^21^	944	63.1	73.2		100		3.2	26.3	10.1	911	62.9	69.9		100		5.4	25.1	10.5
Serruys (2001)^26^	600	61	77		19	45	5	44	6	605	61	76		16	45	5	42	5
Zhang (2003)^25^	235	65	78		32	72			9.4	231	65	78		28	70			6.9

CABG indicates coronary artery bypass grafting; COPD, chronic obstructive pulmonary disease; MI, myocardial infarction; PCI, percutaneous coronary intervention; and PVD, peripheral vascular disease.

### Seattle Angina Questionnaire‐AF

**Figure 1:**
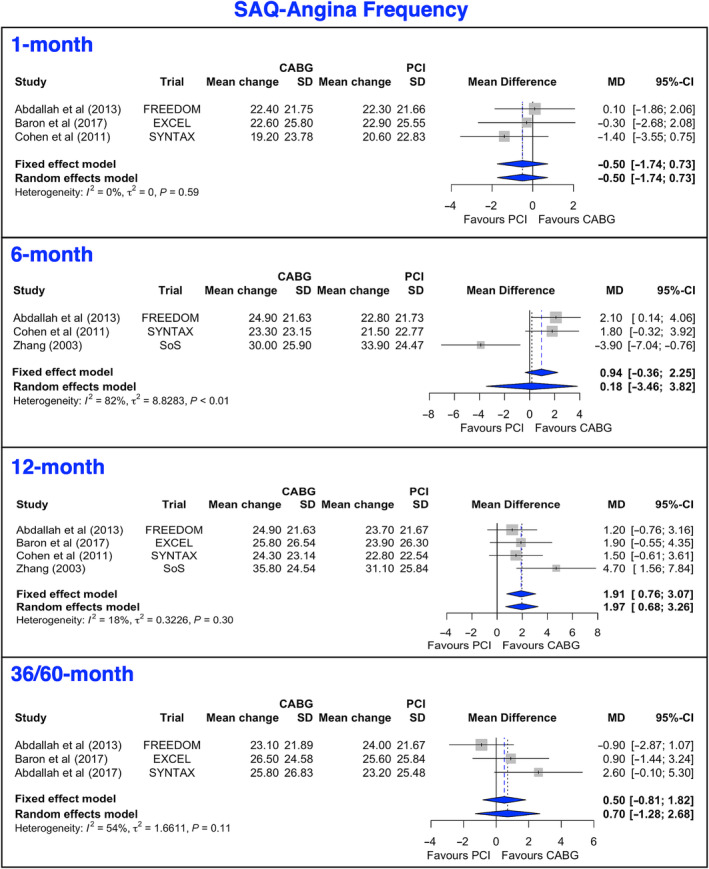
Forest plots showing pooled weighted mean differences of angina frequency scores with 1, 6, 12, and 36/60 months follow‐up data for SAQ‐AF.^20^, ^22^, ^24^, ^25^, ^27^ CABG indicates coronary artery bypass grafting; MD, mean difference; PCI, percutaneous coronary intervention; and SAQ‐AF, Seattle Angina Questionnaire‐Angina Frequency.

The primary outcome, SAQ‐AF, was reported in all 5 trials at baseline and 12 months, and in 3 trials at 1, 6, and 36 to 60 months. The mean change from baseline to 12 months and at latest follow‐up (36–60 months) after both PCI and CABG was >22 points (95% CI, 21–25.6) (Figure 1, ^20^, ^22^, ^24^, ^25^, ^27^; Figure S3, ^20^, ^21^, ^22^, ^23^, ^24^, ^27^). Mean gains in SAQ‐AF were similar between PCI and CABG at 1, 6, or 36 to 60 months. At 12‐month follow‐up, the gain in SAQ‐AF was greater after CABG than PCI (difference 1.97 points [95% CI, 0.68–3.26]; *I* ^2^ = 18%]). The 12‐month results were corroborated by the leave‐1‐out analysis (Figure S4, ^20^, ^22^, ^24^, ^27^), and meta‐regression showed that mean gains were not associated with sex, age, diabetes, baseline QoL score, and year of publication (Table 3).

**Table 3:** Meta‐Regression Results for the Primary Outcome

	Estimate (β‐coeff)	95% CI	*P* value
SAQ‐AF			
Age	−0.4090	−1.4290 to 0.6109	0.43
Diabetes	−0.0192	−0.0567 to 0.0183	0.32
Sex (M)	0.1584	−0.3473 to 0.6642	0.53
Year	−0.21	−0.4847 to 0.0733	0.15
Baseline score	−0.2095	−0.4261 to 0.0070	0.06
SAQ‐QoL			
Age	−0.3075	−1.3374 to 0.7224	0.56
Diabetes	−0.0025	−0.0473 to 0.0423	0.91
Sex (M)	−0.0197	−0.5535 to 0.5141	0.94
Year	−0.0819	−0.3932 to 0.2294	0.61
Baseline score	0.0364	−0.2710 to 0.3438	0.82
SAQ‐PL			
Age	−1.3899	−2.1846 to −0.5952	0.0006
Diabetes	0.0304	−0.0438 to 0.1045	0.42
Sex (M)	−0.4745	−1.2675 to 0.3186	0.24
Year	−0.2784	−0.7327 to 0.1759	0.23
Baseline score	−0.1208	−0.7016 to 0.4601	0.68
EQ‐5D			
Age	0.0030	−0.0222 to 0.0281	0.82
Diabetes	−0.0010	−0.0013 to −0.0008	<0.001
Sex (M)	0.0086	0.0037–0.0135	<0.001
Year	−0.0014	−0.0090 to 0.0063	0.72
Baseline score	−0.4019	−1.4036 to 0.5998	0.43
SF‐PC			
Age	−0.1832	−0.5424 to 0.1759	0.32
Diabetes	−0.0756	−0.1837 to 0.0326	0.17
Sex (M)	−0.4083	−0.9101 to 0.0936	0.11
Year	−0.0428	−0.1775 to 0.0919	0.53
Baseline score	−0.3628	−0.7799 to 0.0542	0.09
SF‐MC			
Age	−0.1602	−0.5139 to 0.1934	0.37
Diabetes	−0.0503	−0.1766 to 0.0759	0.43
Sex (M)	−0.1694	−0.7653 to 0.4265	0.58
Year	−0.0552	−0.1645 to 0.0542	0.32
Baseline score	−0.1305	−0.4721 to 0.2112	0.45

AF indicates angina frequency; EQ‐5D, EuroQoL‐5D; MC, mental component; PC, physical component; PL, physical limitation; QoL, quality of life; SAQ, Seattle Angina Questionnaire; and SF‐36, 36‐Item Short Form Health Survey.

### Seattle Angina Questionnaire‐QoL

**Figure 2:**
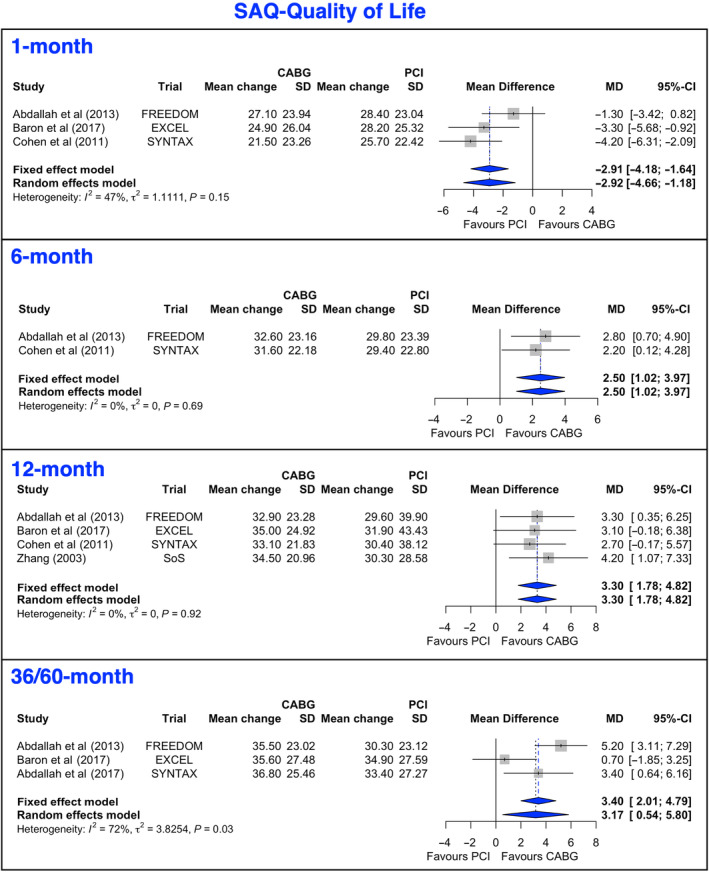
Forest plots showing pooled weighted mean differences of quality of life scores with 1, 6, 12, and 36/60 months follow‐up data for SAQ‐QoL.^20^, ^22^, ^24^, ^25^, ^27^ CABG indicates coronary artery bypass grafting; MD, mean difference; PCI, percutaneous coronary intervention; and SAQ‐QoL, Seattle Angina Questionnaire‐Quality of Life.

The mean change from baseline to 12 months and at 36 to 60 months after both PCI and CABG was >29 points (Figure 2, ^20^, ^22^, ^24^, ^25^, ^27^; Figure S5, ^20^, ^21^, ^22^, ^23^, ^24^, ^27^). At 1 month, the mean gains in SAQ‐OL favored PCI, while at 6, 12, and 36/60 months, they favored CABG. These findings were corroborated by the leave‐1‐out analyses at 12 months (Figure S6, ^20^, ^22^, ^24^, ^27^). The meta‐regression results showed that mean gains in SAQ‐QoL were not significantly associated with sex, age, diabetes, baseline QoL score, and year of publication (Table 3).

### Seattle Angina Questionnaire‐PL

**Figure 3:**
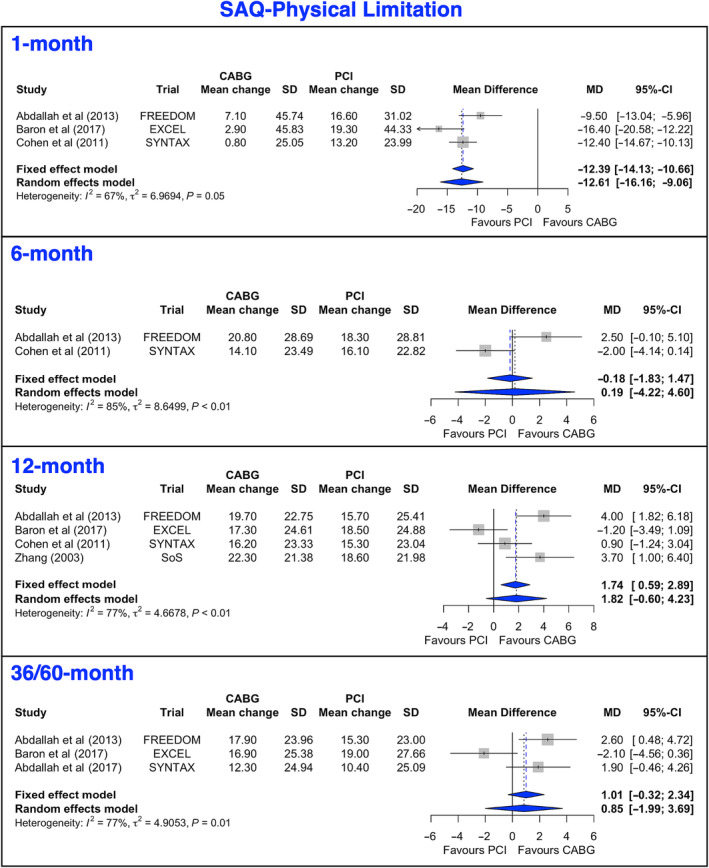
Forest Plots showing pooled weighted mean differences of physical limitation scores with 1, 6, 12, and 36/60 months follow‐up data for SAQ‐PL.^20^, ^22^, ^24^, ^25^, ^27^ CABG indicates coronary artery bypass grafting; MD, mean difference; PCI, percutaneous coronary intervention; and SAQ‐PL, Seattle Angina Questionnaire‐Physical Limitation.

The mean change from baseline to 12 months and at 36 to 60 months after both PCI and CABG was >15 points (Figure 3, ^20^, ^22^, ^24^, ^25^, ^27^; Figure S7, ^20^, ^21^, ^22^, ^23^, ^24^, ^27^). For SAQ‐PL, the mean gain favored PCI at 1 month, with no differences at 6, 12, or 36/60 months. Meta‐regression demonstrated that the mean gain in SAQ‐PL at 12 months was inversely associated with the age of patients but not related to sex, age, diabetes, baseline QoL score, and year of publication (Table 3). In the leave‐1‐out analysis, when the EXCEL (Evaluation of XIENCE versus Coronary Artery Bypass Surgery for Effectiveness of Left Main Revascularization) trial^24^ was excluded, there was a significantly higher 12‐month mean gain in SAQ‐PL for patients undergoing CABG compared with PCI (Figure S8, ^20^, ^22^, ^24^, ^27^).

Funnel plots and Egger's tests did not show potential publication bias or small‐study effect in any of the SAQ domains analyzed (Figure S9).

### EuroQoL‐5D

**Figure 4:**
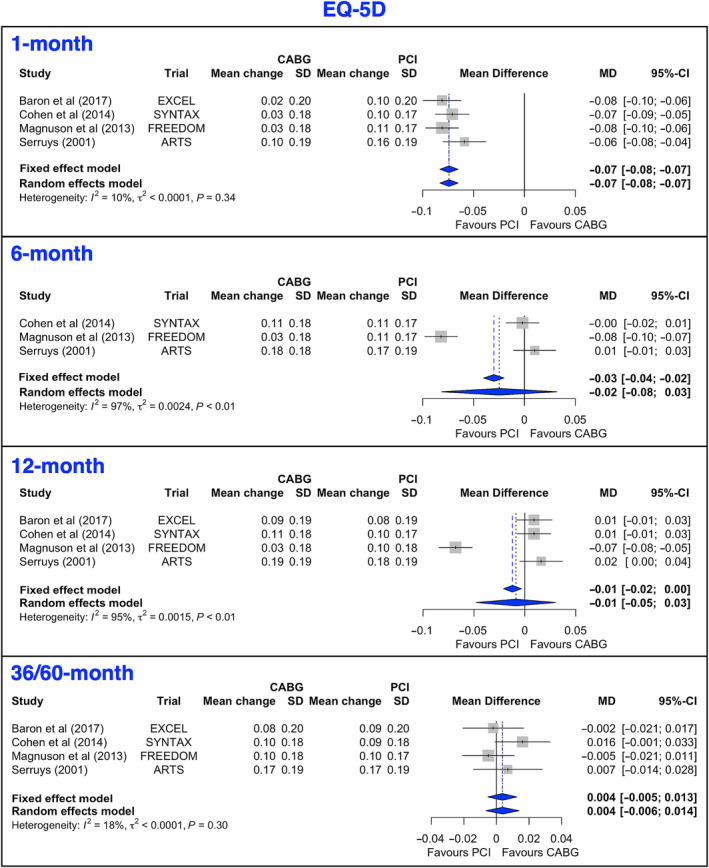
Forest plots showing pooled weighted mean differences of generic quality of life scores with 1, 6, 12, and 36/60 months follow‐up data for EQ‐5D.^21^, ^23^, ^24^, ^26^ CABG indicates coronary artery bypass grafting; EQ‐5D, EuroQoL‐5D; MD, mean difference; and PCI, percutaneous coronary intervention.

The mean gain in EQ‐5D was pooled from available data in 4 trials. At 1 month, the mean gain favored PCI, but there were no significant differences at 36/60 months. At 6 and 12 months, the mean gains favored PCI in fixed‐effect analysis but not in random‐effect analysis (Figure 4, ^21^, ^23^, ^24^, ^26^; Figure S10, ^20^, ^21^, ^22^, ^23^, ^24^, ^27^). In the leave‐1‐out analysis for the 12‐month estimate, when the FREEDOM (Future Revascularization Evaluation in Patients with Diabetes Mellitus: Optimal Management of Multivessel Disease) trial^20^ (a trial of only patients with diabetes) was excluded, there was a marginally higher mean gain with CABG compared with PCI at 12 months in the random‐effect analysis (Figure S11, ^22^, ^23^, ^26^, ^27^). The mean gain in EQ‐5D at 12 months was inversely associated with the proportion of patients with diabetes (Table 3). Conversely, a higher proportion of male participants was associated with a higher mean gain in the EQ‐5D (Table 3). There was no evidence of publication bias or small‐study effect (Figure S12).

### Short‐Form 12/36 Physical and Mental Component

**Figure 5:**
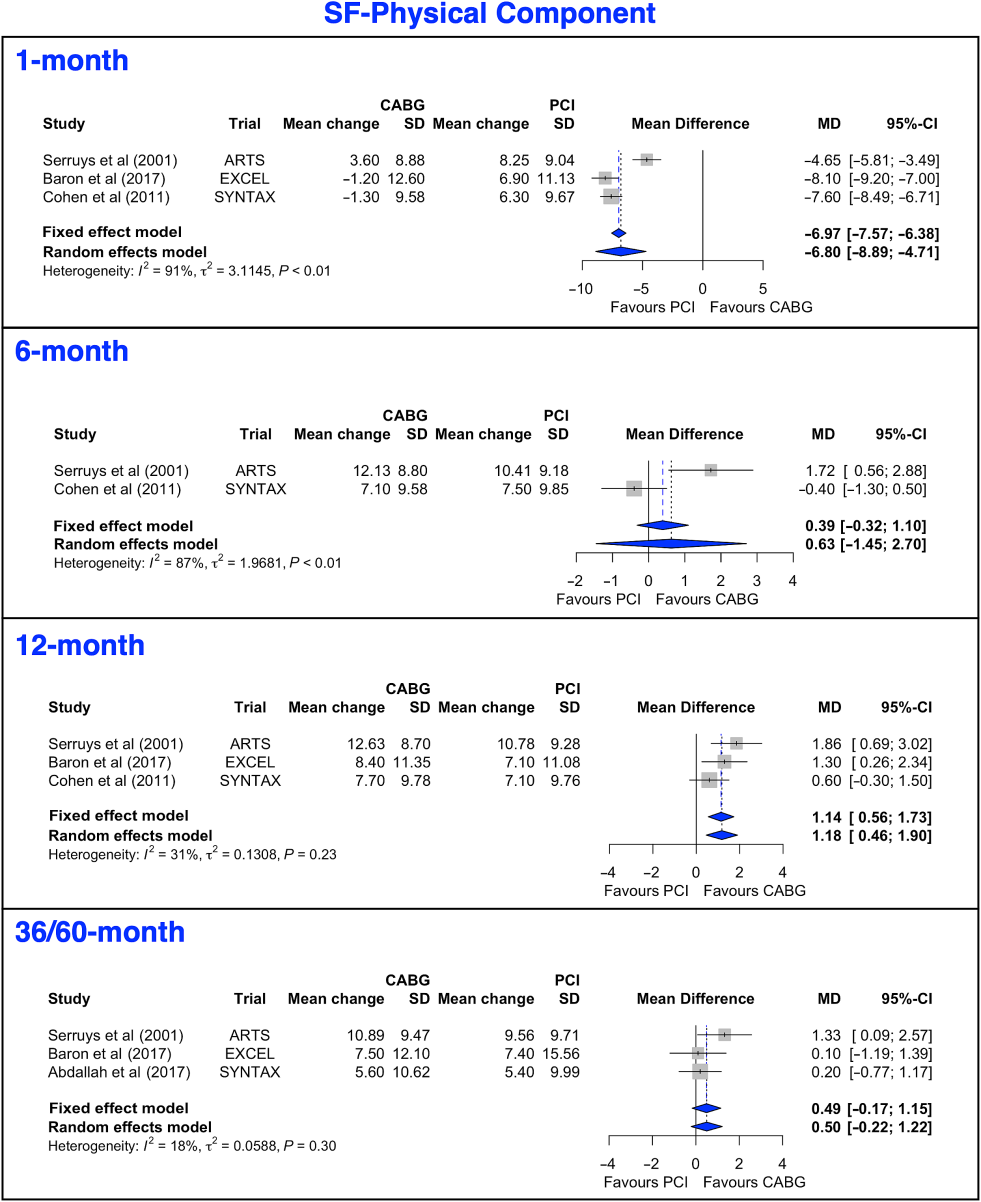
Forest plots showing pooled weighted mean differences of the physical quality of life scores with 1, 6, 12, and 36/60 months follow‐up data for SF‐PC.^22^, ^24^, ^26^, ^27^ CABG indicates coronary artery bypass grafting; MD, mean difference; PCI, percutaneous coronary intervention; and SF‐PC, Short Form Physical Component.

**Figure 6:**
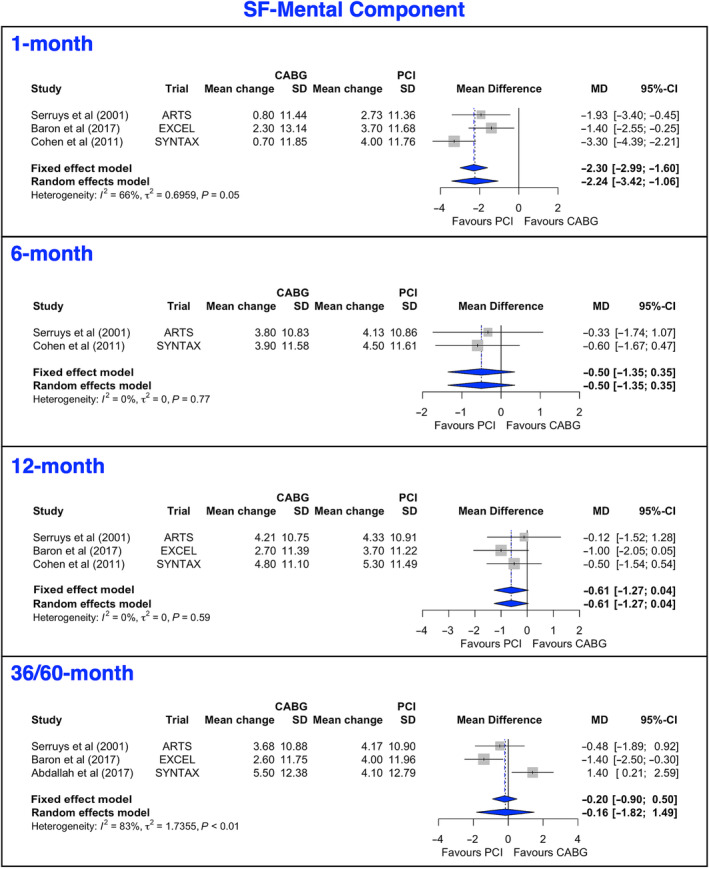
Forest plots showing pooled weighted mean differences of mental quality of life scores with 1, 6, 12, and 36/60 months follow‐up data for SF‐MC.^2^, ^22^, ^24^, ^26^ CABG indicates coronary artery bypass grafting; MD, mean difference; PCI, percutaneous coronary intervention; and SF‐MC, Short Form Mental Component.

One trial reported SF‐12 scores and 2 reported SF‐36 scores. The mean gains in PCS scores (Figure 5, ^22^, ^24^, ^26^, ^27^; Figure S13, ^22^, ^23^, ^24^, ^26^, ^27^) favored PCI at 1 month, favored CABG at 12 months, and were not statistically different at 6 or 36 to 60 months. The leave‐1‐out analysis for PCS at 12 months is presented in Figure S14.^22^, ^24^, ^26^ The Egger's test revealed significant asymmetry of the funnel plot, suggesting potential publication bias (Figure S15). The main gains in MCS scores (Figure 6, ^22^, ^24^, ^26^, ^27^ and Figure S16, ^22^, ^23^, ^24^, ^26^, ^27^) favored PCI at 1 month but were not statistically different at 6, 12, or 36 to 60 months. The leave‐1‐out analysis at 12 months for MCS is presented in Figure S17.^22^, ^24^, ^26^ There was no evidence of publication bias or small‐study effect (Figure S18).

## DISCUSSION

For patients with CAD, whether and how to undergo revascularization are important treatment considerations. Current guidelines emphasize the importance of shared decision‐making,^5^ for which describing differences in QoL improvement is critical. The present meta‐analysis extends prior work describing the differences in clinical events to explore the differences in improvement in QoL, a critically important outcome for patients, following CABG and PCI from 5 RCTs enrolling 10 760 patients. Overall, both CABG and PCI provided marked long‐term improvements in patients' disease‐specific and generic QoL compared with baseline, as shown by the mean changes that markedly exceeded the minimally clinically important differences (eg, 5–8 points in the SAQ).^28^

In general, we found greater improvements in QoL with PCI at 1 month and either similar or better improvements in QoL at 12 months with CABG. Differences between procedures were larger for disease‐specific physical symptoms, such as angina relief, but were also evident for physical function and generic health status. At the latest follow‐up (36–60 months) there were no significant differences in either disease‐specific or generic QoL between the procedures except for a small gain in SAQ‐QoL favoring CABG (≈3 points). Of note, 2 out of 3 studies ended follow‐up at 36 months, and only 1 study reported data out to 60 months. Describing the trajectory of recovery in more depth, by having complete data at 36 and 60 months (and ideally up to 10 years) would provide more clarity on the differences in longer‐term outcomes between PCI and CABG.

A major advantage of our study compared with previous meta‐analyses is that we included only high‐quality data from RCTs with short‐ and long‐term follow‐up. Previous systematic reviews have included nonrandomized studies, which can bias estimates due to residual confounding, ascertainment bias, and heterogeneity in follow‐up methods.^7^, ^29^

Overall, most QoL metrics favored PCI in the first month. The findings of greater improvement in QoL at 1 month after PCI reflects the lesser invasive nature of the PCI procedure compared with CABG, including fewer procedural complications, a shorter length of stay, and faster recovery. In contrast, most patients undergoing CABG experience sternal wound and graft site pain for the first few weeks after surgery and undergo extensive cardiac rehabilitation. The early relative benefit in QoL after PCI versus CABG was no longer apparent at 6 months. At 12 months, there was greater improvement in 2 of the 3 disease‐specific QoL measures of angina‐related symptoms and in 1 of the 3 generic QoL measures with CABG compared with PCI. However, at 36 to 60 months, there were minimal differences in most of the disease‐specific and generic QoL measures between PCI and CABG. In this regard, the most fundamental finding from the present study is that both procedures led to marked improvements in QoL, and that any differences between procedures were modest in comparison. For example, the SAQ‐AF improved from baseline to 12 months by ≈25 to 30 points after both procedures, with a mean weighted difference of 2 points favoring CABG.

There were no differences between CABG and PCI in physical limitations at 12 months and 36 to 60 months, whereas there were differences favoring PCI at 1 month. This could be explained by the more invasive approach with surgery and the need for a longer recovery period.^30^ The surgical recovery from CABG limits many generic aspects of physical mobility, including walking, showering, and participating in activities of daily living in the first few weeks and months, but are typically resolved by 6 months.

Generic QoL as measured by SF‐12 and SF‐36 included a physical and mental component and the EQ‐5D. The PCS favored PCI at 1 month and CABG at 12 months, but these differences were not sustained out to 60 months. Differences in the EQ‐5D and MCS were consistent in favoring PCI at 1 month. Of note, at 12 months the mean gains in EQ‐5D favored PCI in fixed‐effect analysis but not in random‐effect analysis. These results were heavily weighted by the FREEDOM trial that only included patients with diabetes.^5^ In this regard, a recently published analysis from the FAME 3 (Fractional Flow Reserve Versus Angiography for Multivessel Evaluation) trial (that was not available for inclusion in the present meta‐analysis) demonstrated a higher EQ‐5D score after PCI compared with CABG at 1 year.^31^ The early benefit of PCI over CABG for both physical and mental symptoms is likely explained by less postsurgical musculoskeletal pains, faster wound healing, and other postsurgical complications that are more common after CABG. Whether these differences end at 6 months, 1 year, or later, and whether this varies in patients with diabetes versus patients who are not diabetic, deserves further study, though the most recent guidelines^5^ do recommend CABG over PCI for patients with diabetes and multivessel disease. Nonetheless, all 12‐month differences, even if statistically significant, were small, and at long‐term follow‐up there are minimal differences in QoL between PCI and CABG.

A critical challenge in the interpretation of clinical trials is that they typically report mean group effects and meta‐analyses and then summarize these population‐level effects. Thus, the mean between‐group differences reported in this study at 12 months and thereafter were small. This may mask the distribution of patients who benefited greatly, had neutral outcomes, or were harmed by one of the treatments. For future studies, we suggest additional outcomes reported in responder analyses, for example, the proportions of patients who improve (or deteriorate) by clinically important magnitudes on a PRO.^32^ Without these data, the interpretation of the clinical importance of the observed mean differences is difficult to understand and communicate to patients and providers alike.

### Limitations

The results of the present meta‐analysis must be interpreted within the context of common limitations in procedural randomized trials, including lack of blinding, rigorous inclusion/exclusion criteria that may limit generalizability, loss to follow‐up for QoL outcomes, and the potential expertise of participating centers. Moreover, it is noteworthy that “immortal time” bias could be present because QoL questionnaires can be completed only by participants who are alive and free of major impairments that would prevent their completion. This also applies to patients who have had a major cerebrovascular event, such as stroke, which would prevent completion of follow‐up questionnaires. Overall, the proportion of participants who completed baseline to follow‐up data at 1 year was 80%.

In addition, we did not have patient‐level data from these studies to adjust for patient‐level characteristics more rigorously and to explore potential heterogeneity of relative treatment benefits by these factors or to conduct responder analyses to help interpret the mean differences between groups throughout follow‐up or to jointly model the potential impact of death. In trials where deaths favor 1 group, the patients who die are more likely to have worse health status; thus there are benefits of jointly modeling the outcomes so as not to underestimate the benefits of a treatment that improves survival.^33^ Given the similar mortality after PCI and CABG at 12 months from most RCTs, this was likely not of major consequence to the present study at this time point but may have affected later outcomes in patients with extensive (non‐left main‐related) CAD in whom survival favors CABG. We were also not able to evaluate longer‐term time points (eg, 10 years) due to the absence of studies including QoL data at this follow‐up duration. To provide an estimate beyond 1 year, we combined 36‐ and 60‐month assessments because there were not enough studies to perform a meta‐analysis at each individual time point. Finally, a significant limitation of the collective trial population was that it was predominantly white and male. Many studies did not report race or ethnicity, and those that did reported race and ethnicity consistent with country‐specific census categories. Additional detailed information on the impact of race and ethnicity on QOL outcomes was not available in the aggregate data of the included studies. This limits the generalizability of the study findings to the participants represented in the included studies.

## CONCLUSIONS

Improvements in QoL and symptoms are essential components of patient‐centered care.^34^ In the present systematic review and meta‐analysis, disease‐specific and generic QoL of patients with complex CAD undergoing revascularization favored PCI at 1 month and were similar or favored CABG at 12 months. However, QoL improved markedly after both procedures, and any late (36–60 months) mean differences between treatments were relatively small. A better understanding of the variables that affect intermediate and long‐term differences in different QoL metrics after CABG versus PCI is important for allowing individual patients to make informed decisions about revascularization strategy.

## Sources of Funding

R.M.C. receives funding from NHLBI (R01HL152021), NINDS (R01NS123639), and NHLBI (R01HL161458). M.G. receives funding from NHLBI (R01HL152021) and NINDS (R01NS123639).

## Disclosures

Dr Stone has received speaker honoraria from Pulnovo, Infraredx; has served as a consultant to Valfix, TherOx, Robocath, HeartFlow, Ablative Solutions, Vectorious, Miracor, Neovasc, Abiomed, Ancora, Elucid Bio, Occlutech, CorFlow, Apollo Therapeutics, Impulse Dynamics, Vascular Dynamics, Shockwave, V‐Wave, Cardiomech, Gore, Amgen; and has equity/options from Ancora, Cagent, Applied Therapeutics, Biostar family of funds, SpectraWave, Orchestra Biomed, Aria, Cardiac Success, Valfix, and Xenter. Dr Stone's daughter is an employee at IQVIA. Institutional disclosure: Dr Stone's employer, Mount Sinai Hospital, receives research support from Abbott, Bioventrix, Cardiovascular Systems Inc, Phillips, Biosense‐Webster, Shockwave, Vascular Dynamics, and V‐wave. Dr Spertus owns the copyright to the Seattle Angina Questionnaire, Kansas City Cardiomyopathy Questionnaire, and Peripheral Artery Questionnaire. He provides consultative services to Bayer, Bristol Myers Squibb, Merck, Novartis, and Janssen on patient‐reported outcomes and has a research grant from Abbott Vascular for the collection and analyses of PROs. The remaining authors have no disclosures to report.

## Supporting information

Table S1
Figures S1–S18
