# Supplementary material for: Quality of Life After Percutaneous Coronary Intervention Versus Coronary Artery Bypass Grafting
Source: J Am Heart Assoc. 2023 Nov 20;12(22):e030069. doi: 10.1161/JAHA.123.030069 (PMC10727273; doi:10.1161/JAHA.123.030069)

Supplemental Material

Table S1. Search Strategy

**Ovid MEDLINE (ALL – 1946 to May 06, 2021)<br>Searched on May 6, 2021<br>Limited to English language articles published from 2000<br>Limited to RCTs with BMJ's validated study design search filter:<br>https://bestpractice.bmj.com/info/us/toolkit/learn-ebm/study-design-search-filters/**	
**Line #**	**Search**
**1**	Percutaneous Coronary Intervention/
**2**	(percutaneous coronary intervention* or percutaneous coronary revasculari*ation* or percutaneous coronary angioplast* or PCI).tw.
**3**	Stents/ or Drug-Eluting Stents/ or Self Expandable Metallic Stents/
**4**	(stent or stents or stenting or stented).tw.
**5**	Angioplasty, Balloon, Coronary/
**6**	(coronary balloon angioplast* or transluminal coronary balloon dilation or coronary artery balloon dilation or percutaneous transluminal coronary angioplast* or PTCA).tw.
**7**	Atherectomy, Coronary/
**8**	(coronary atherectom* or rotational atherectom*).tw.
**9**	or/1-8
**10**	Coronary Artery Bypass/
**11**	Coronary Artery Bypass, Off-Pump/
**12**	(coronary adj2 (bypass* or graft* or surger*)).tw.
**13**	(CABG or aorticocoronary anastomosis or total arterial revasculari*ation* or multiple arterial revasculari*ation*).tw.
**14**	Internal Mammary-Coronary Artery Anastomosis/
**15**	((right internal mammary artery or RIMA or left internal mammary artery or LIMA or Coronary Internal Mammary Artery or arteria mammaria interna or arteria thoracica interna or internal thoracic artery or mammary internal artery) and (transplant* or graft* or anastomosis)).tw.
**16**	(surgical revasculari*ation* or cardiac muscle revasculari*ation* or coronary revasculari*ation* or heart muscle revasculari*ation* or heart myocardium revasculari*ation* or heart revasculari*ation* or internal mammary arterial anastomosis or internal mammary arterial implant* or internal mammary artery anastomosis or internal mammary artery graft* or internal mammary artery implant* or internal mammary-coronary artery anastomosis).tw.
**17**	or/10-16
**18**	"Quality of Life"/
**19**	(life quality or "quality of life" or HRQOL).tw.
**20**	("SF-36" or "Short Form (36) Health Survey" or "36-Item Short Form Survey" or "short form (SF)-36 health status").tw.
**21**	("SF-12" or " Short Form (12) Health Survey" or "12-Item Short Form Survey" or "short form (SF)-12 health status").tw.
**22**	(PROMIS or Patient-Reported Outcomes Measurement Information System).tw.

**23**	(Seattle Angina Questionnaire or SAQ).tw.
**24**	Walk Test/
**25**	(walk test* or functional status).tw.
**26**	Depression/
**27**	(depression* or depressive*).tw.
**28**	Depressive Disorder/
**29**	melancholia*.tw.
**30**	Depressive Disorder, Major/
**31**	involutional paraphrenia*.tw.
**32**	Depressive Disorder, Treatment-Resistant/ or Dysthymic Disorder/
**33**	dysthymic disorder*.tw.
**34**	Anxiety/
**35**	(anxiet* or anxious* or hypervigilance or nervousness).tw.
**36**	Neuralgia/
**37**	(neuralgia* or neuropathic pain* or neurodynia* or nerve pain*).tw.
**38**	Mobility limitation/
**39**	physical function*.tw.
**40**	((mobility or ambulatory or walk*) adj2 (limit* or difficult*)).tw.
**41**	exp Cognitive Dysfunction/
**42**	(cogniti* impair* or cogniti* dysfunction* or cogniti* decline* or neurocognitive disorder* or mental deterioration* or brain fog* or cognitiv* defect*).tw.
**43**	exp Angina Pectoris/ or (angina* or stenocardia* or angor pectoris).tw.
**44**	or/18-43
**45**	9 and 17 and 44
**46**	limit 45 to yr="2010 -Current"
**47**	"randomized controlled trial".pt.
**48**	(random* or single blind* or double blind* or triple blind*).ti,ab.
**49**	(retraction of publication or retracted publication).pt.
**50**	or/47-49
**51**	(animals not humans).sh.
**52**	((comment or editorial or meta-analysis or practice-guideline or review or letter) not "randomized controlled trial").pt.
**53**	(random sampl* or random digit* or random effect* or random survey or random regression).ti,ab. not "randomized controlled trial".pt.
**54**	50 not (51 or 52 or 53)
**55**	45 and 54

Figure S1. Preferred Reporting Items for Systematic Reviews and Meta-Analyses (PRISMA) flow chart

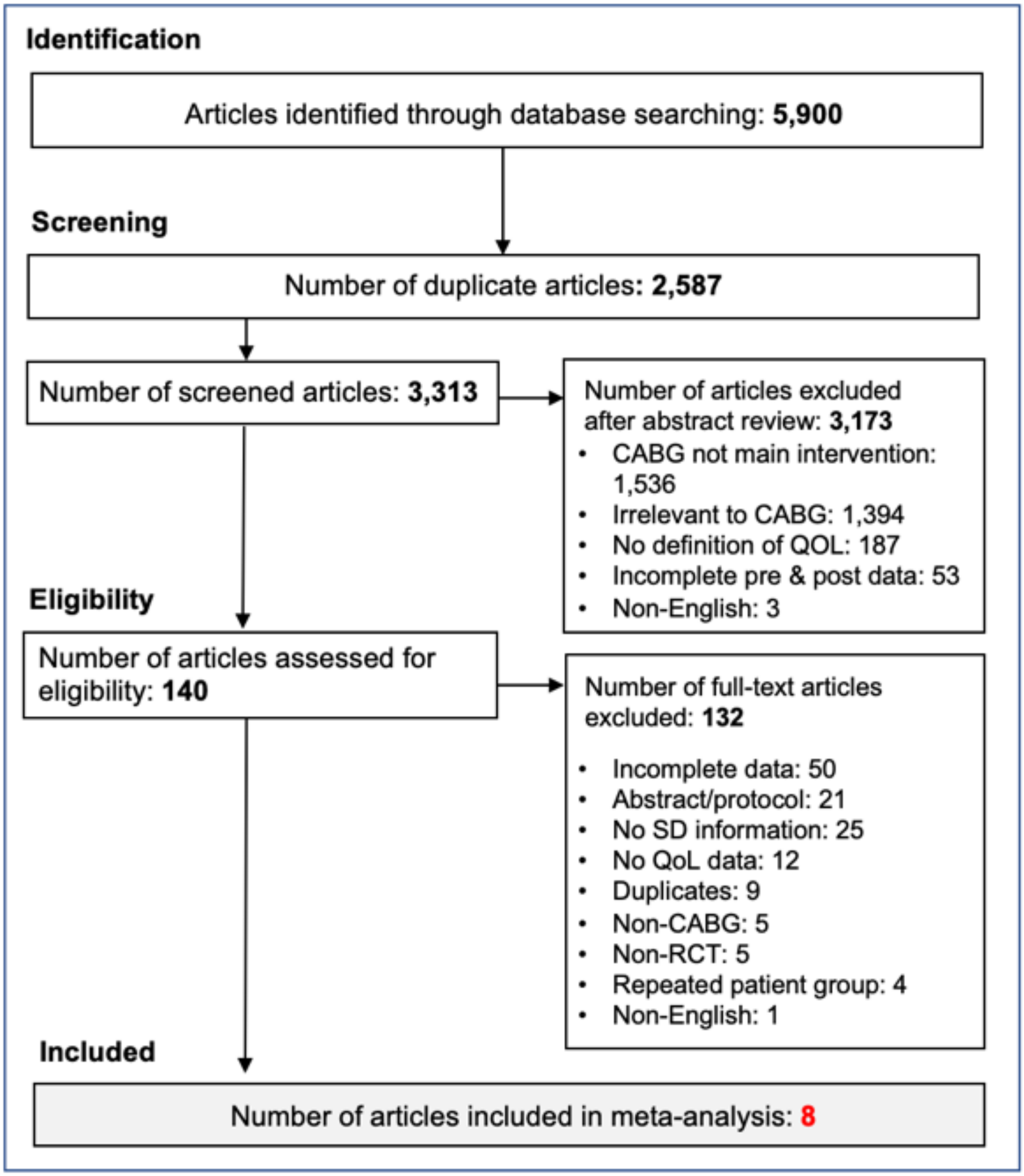

Figure S2. Risk of bias assessment

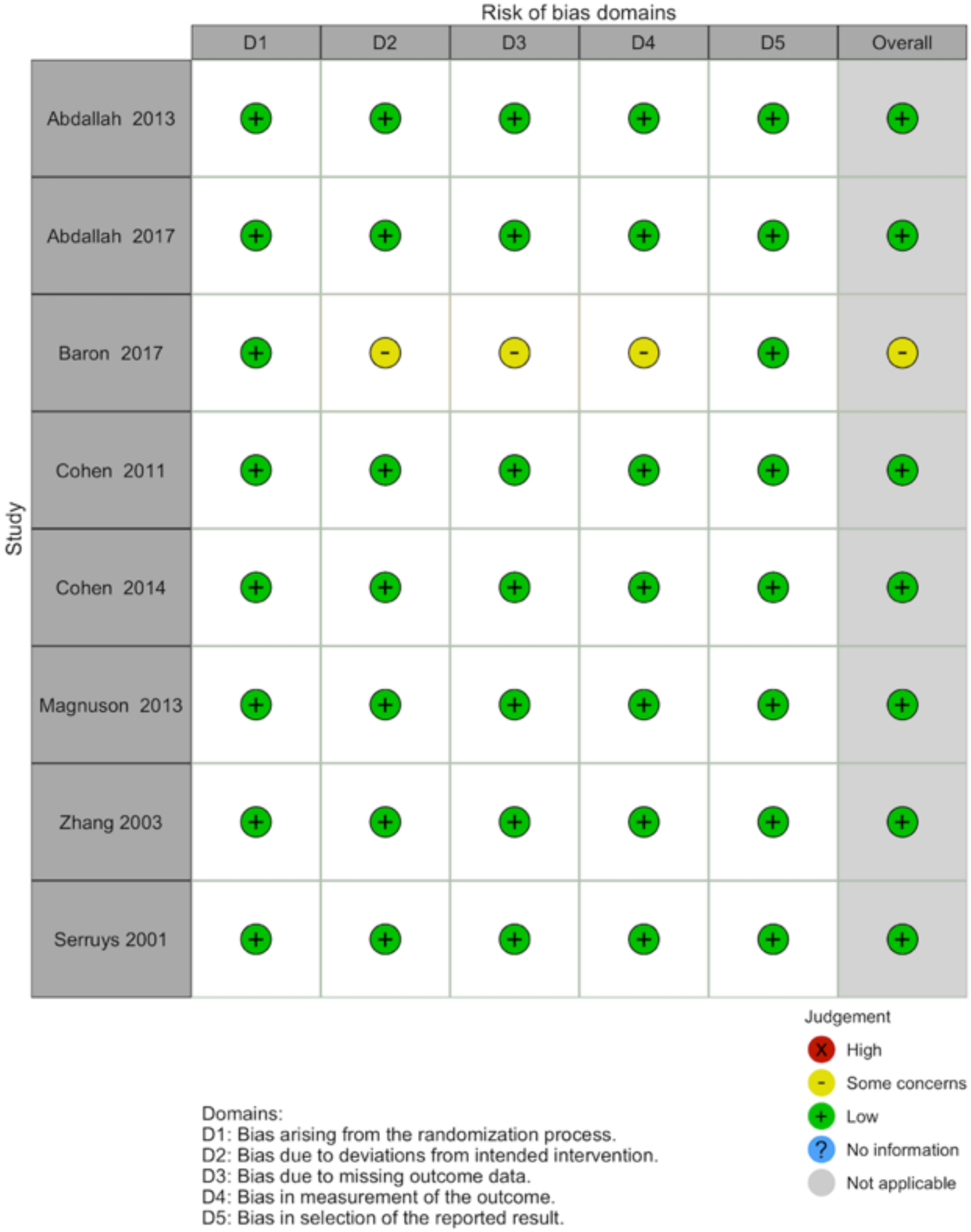

Figure S3. Mean gains in Seattle Angina Questionnaire-Angina Frequency (SAQ-AF) during the follow-up.

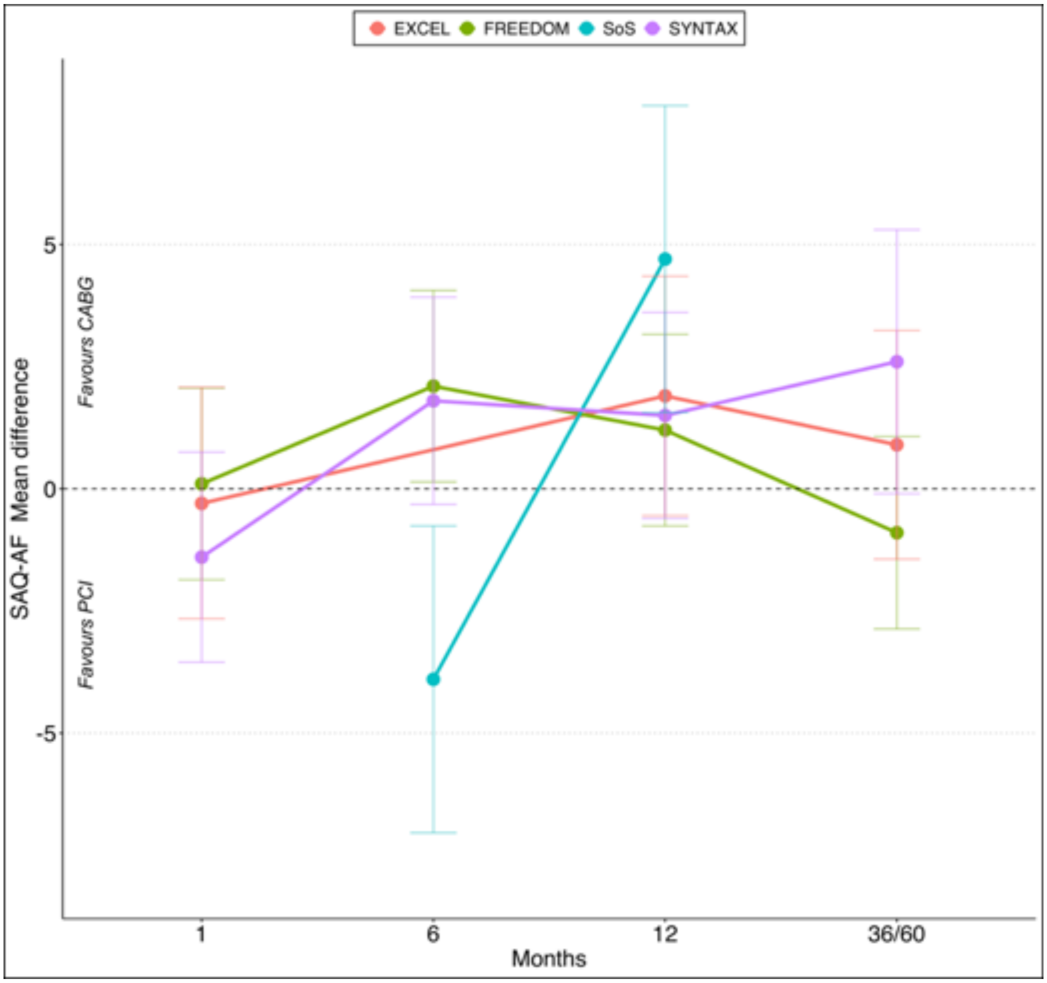

CABG, coronary artery bypass grafting; EXCEL, Evaluation of XIENCE versus Coronary Artery Bypass Surgery for Effectiveness of Left Main Revascularization; FREEDOM, Future Revascularization Evaluation in Patients with Diabetes Mellitus: Optimal Management of Multivessel Disease; PCI, percutaneous coronary intervention; SAQ-AF, Seattle Angina Questionnaire-Angina Frequency; SoS, Stent or Surgery; SYNTAX, Synergy between PCI with TAXUS and Cardiac Surgery.

Figure S4. Leave one out analysis for the Seattle Angina Questionnaire-Angina Frequency at 12 months.

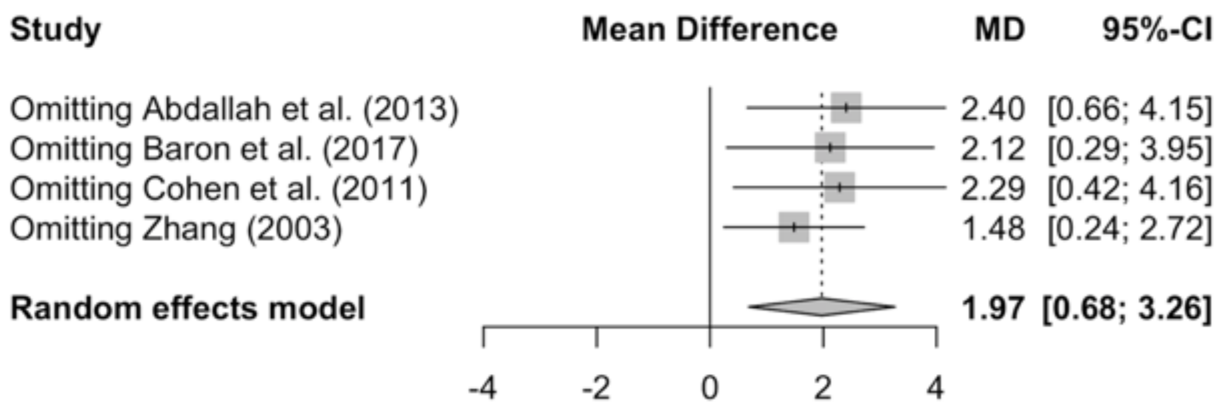

CI, confidence interval; MD, mean difference.

Figure S5. Mean gains in Seattle Angina Questionnaire-Quality of life (SAQ-QoL) during the follow-up.

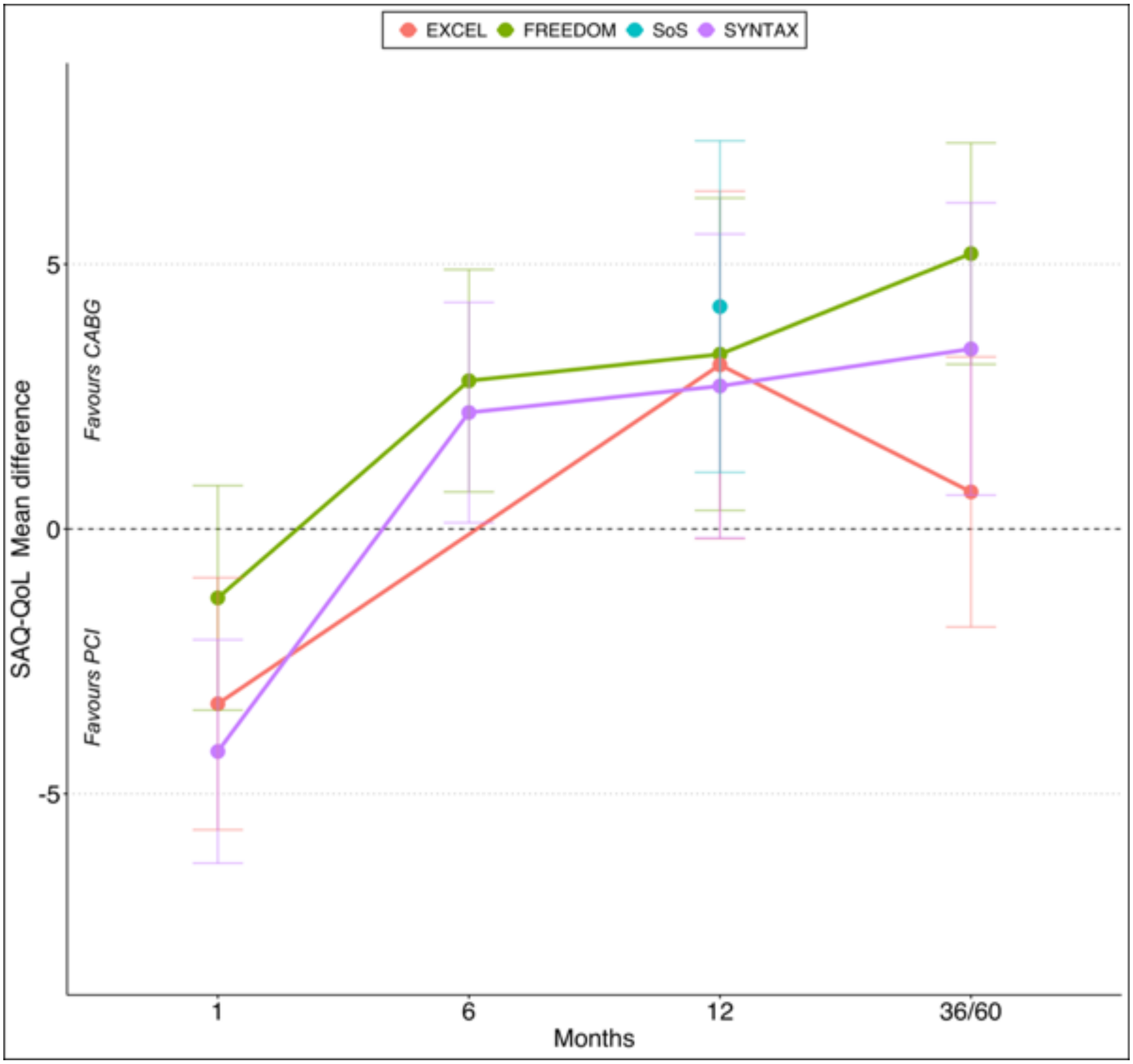

CABG, coronary artery bypass grafting; EXCEL, Evaluation of XIENCE versus Coronary Artery Bypass Surgery for Effectiveness of Left Main Revascularization; FREEDOM, Future Revascularization Evaluation in Patients with Diabetes Mellitus: Optimal Management of Multivessel Disease; PCI, percutaneous coronary intervention; SAQ-QoL, Seattle Angina Questionnaire-Quality of life; SoS, Stent or Surgery; SYNTAX, Synergy between PCI with TAXUS and Cardiac Surgery.

Figure S6. Leave-one-out analysis for the Seattle Angina Questionnaire-Quality of Life at 12 months.

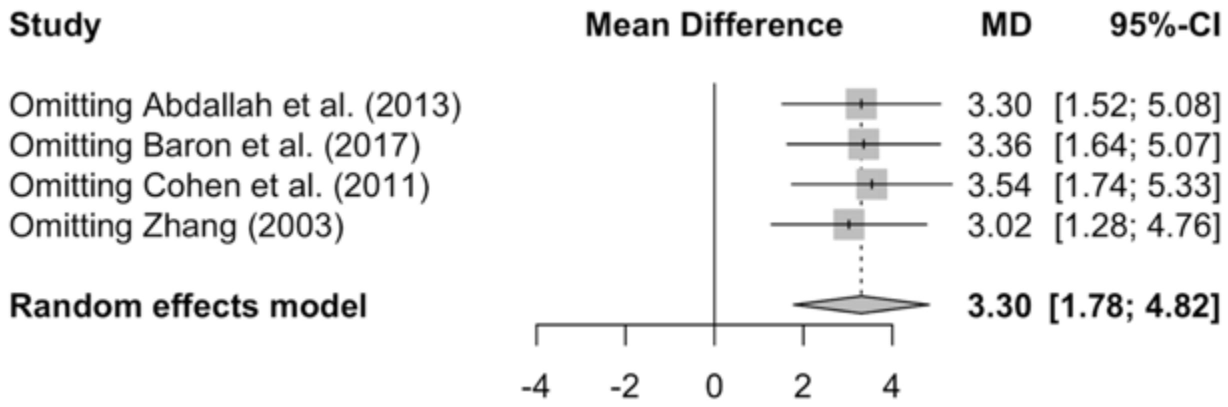

CI, confidence interval; MD, mean difference.

Figure S7. Mean gains in Seattle Angina Questionnaire-Physical Limitations (SAQ-PL) during the follow-up.

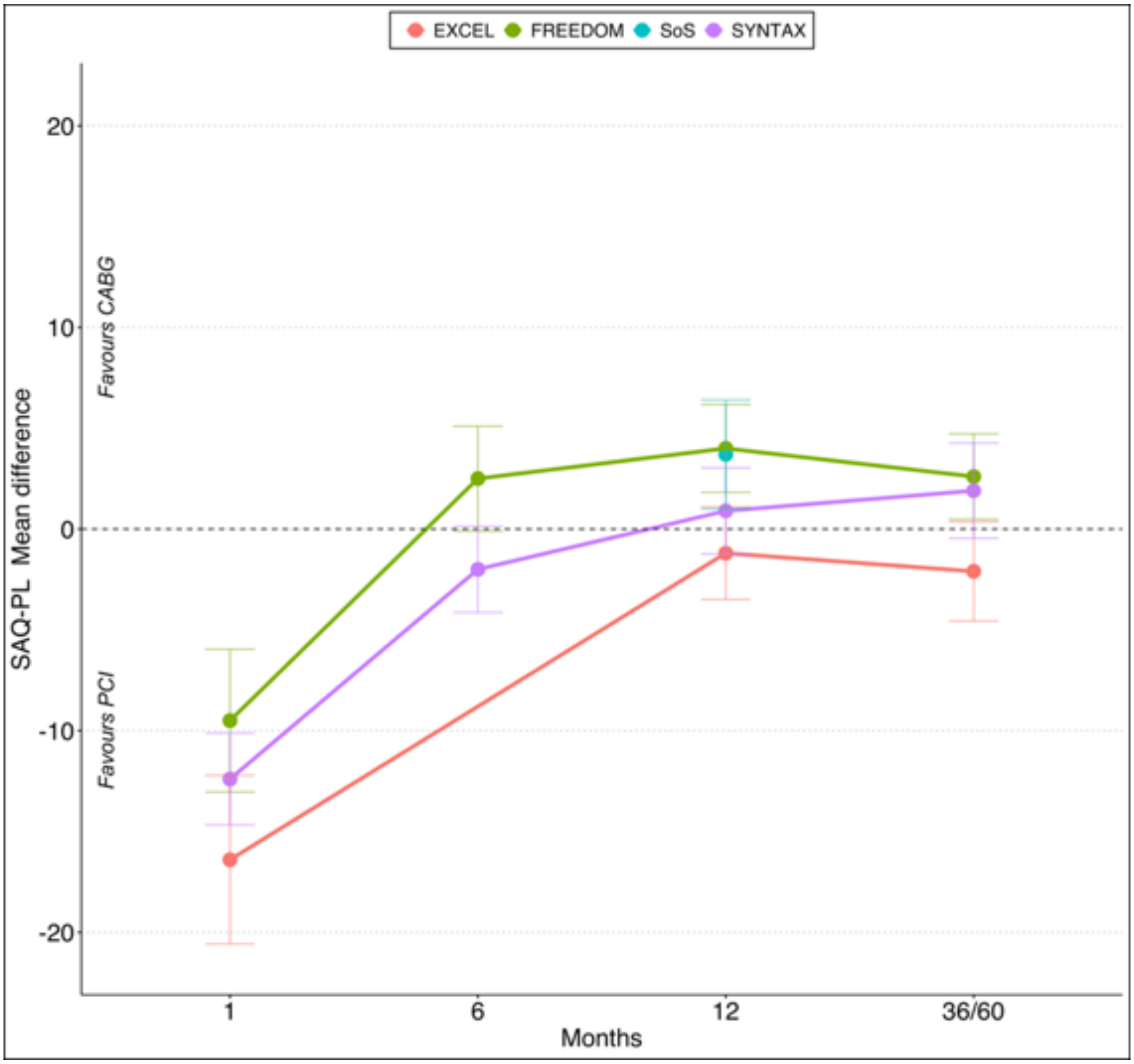

CABG, coronary artery bypass grafting; EXCEL, Evaluation of XIENCE versus Coronary Artery Bypass Surgery for Effectiveness of Left Main Revascularization; FREEDOM, Future Revascularization Evaluation in Patients with Diabetes Mellitus: Optimal Management of Multivessel Disease; PCI, percutaneous coronary intervention; SAQ-PL, Seattle Angina Questionnaire-Physical Limitations; SoS, Stent or Surgery; SYNTAX, Synergy between PCI with TAXUS and Cardiac Surgery.

Figure S8. Leave-one-out analysis for the Seattle Angina Questionnaire- Physical Limitations

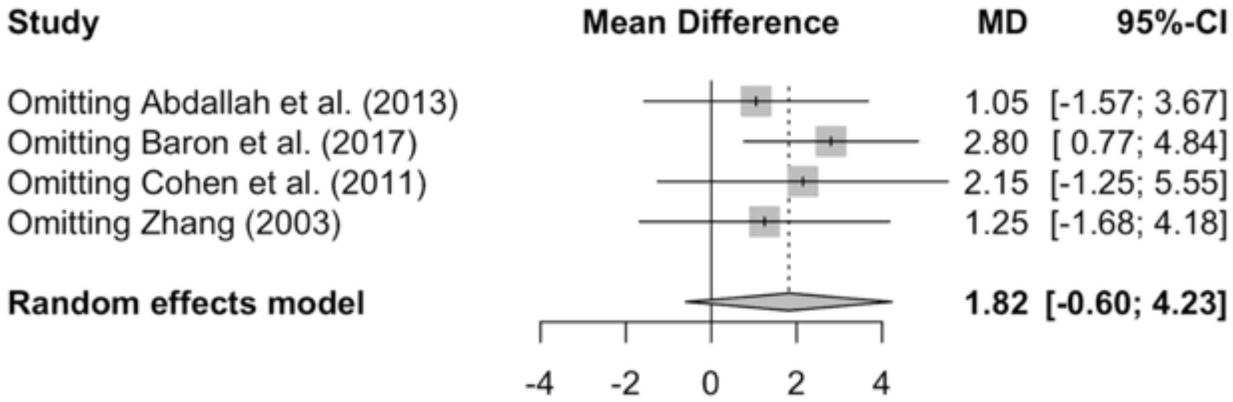

CI, confidence interval; MD, mean difference.

Figure S9. Funnel plots for Seattle Angina Questionnaire-Angina Frequency (SAQ-AF), Quality of Life (SAQ-QoL) and Physical Limitations (SAQ-PL).

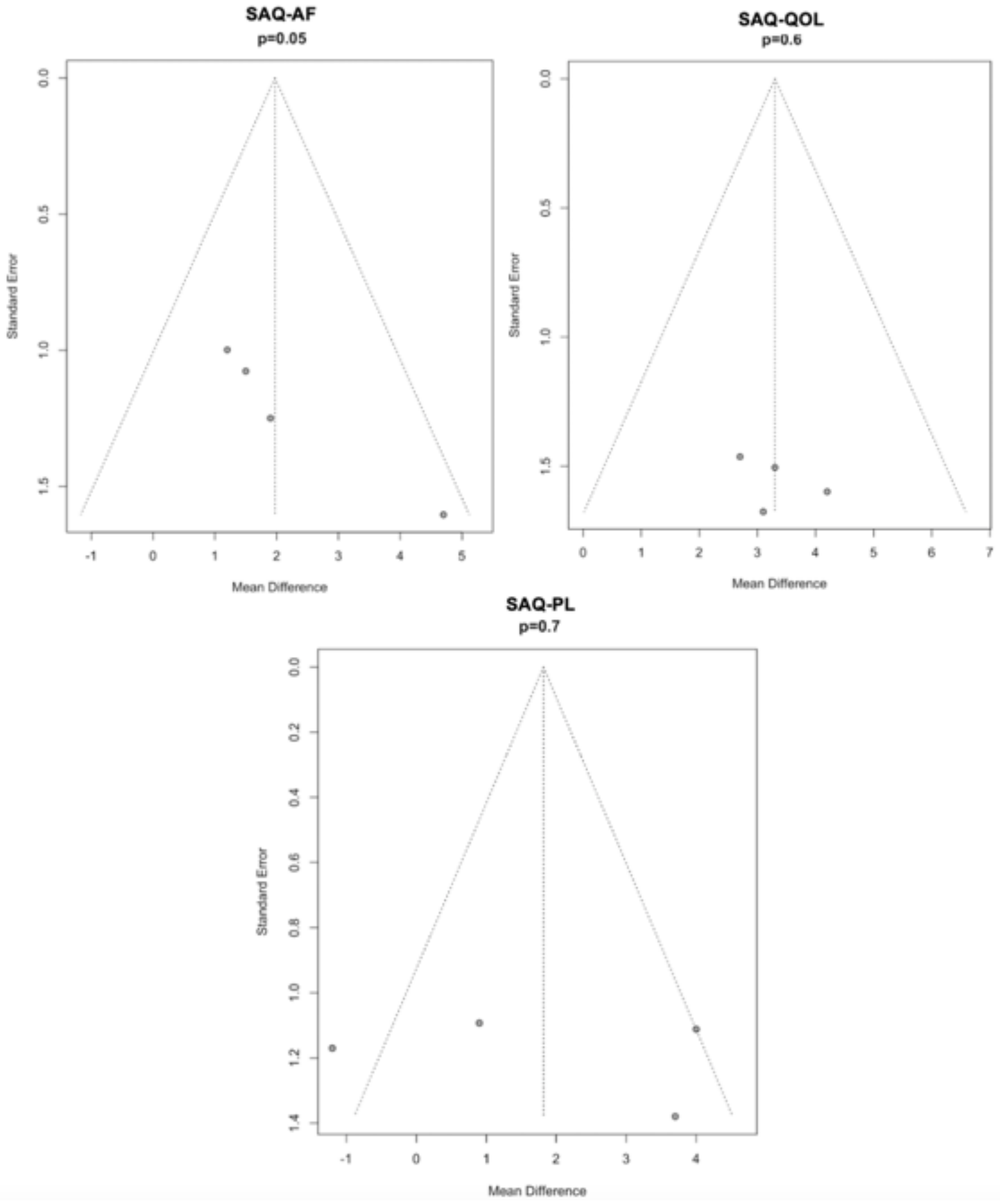

Figure S10. Mean gains in Euro-QoL-5D (EQ-5D) during the follow-up.

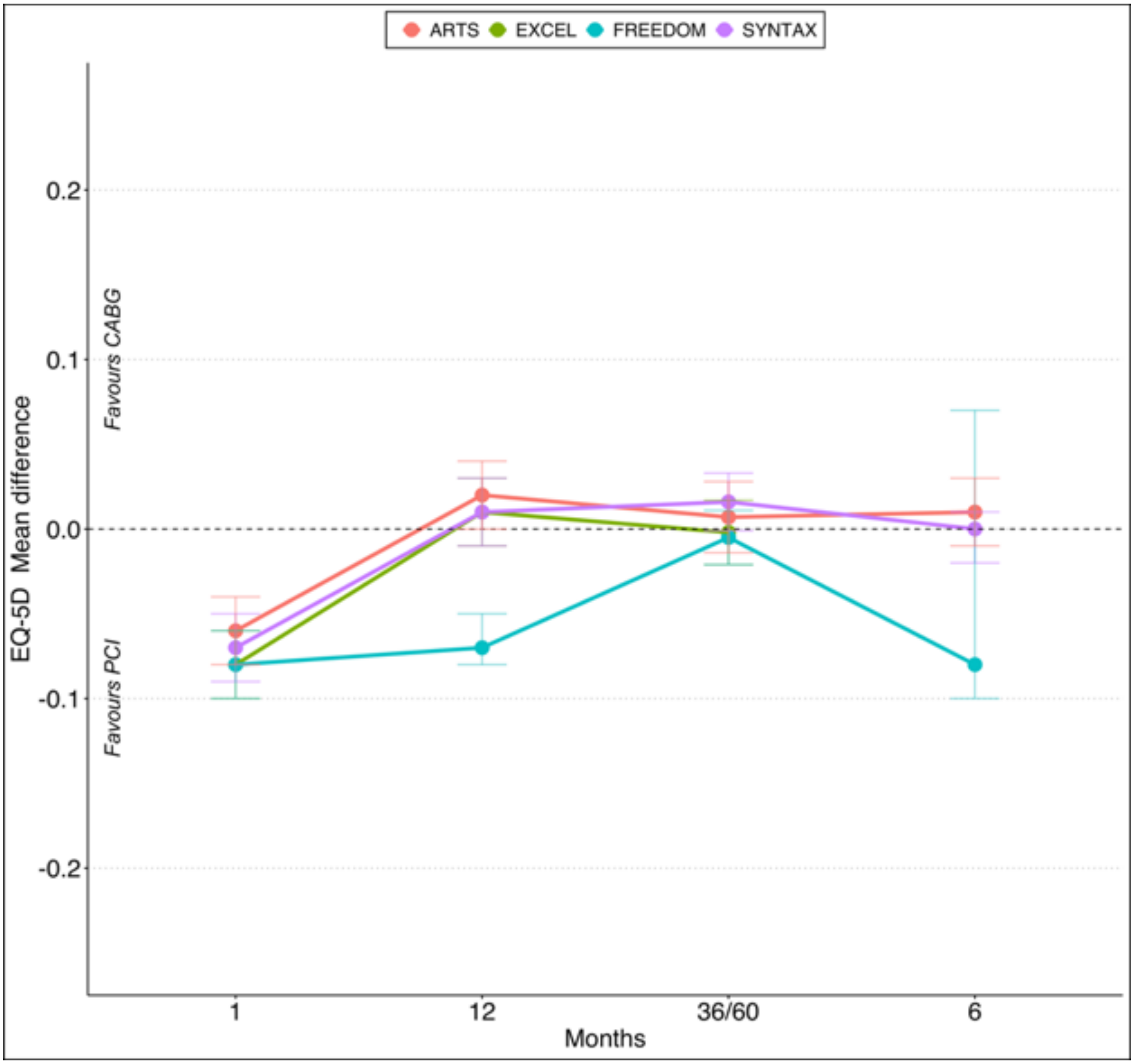

CABG, coronary artery bypass grafting; EXCEL, Evaluation of XIENCE versus Coronary Artery Bypass Surgery for Effectiveness of Left Main Revascularization; FREEDOM, Future Revascularization Evaluation in Patients with Diabetes Mellitus: Optimal Management of Multivessel Disease; PCI, percutaneous coronary intervention; EQ-5D, Euro-QoL-5D; SoS, Stent or Surgery; SYNTAX, Synergy between PCI with TAXUS and Cardiac Surgery.

Figure S11. Leave-one-out analysis for the EuroQoL-5D at 12 months

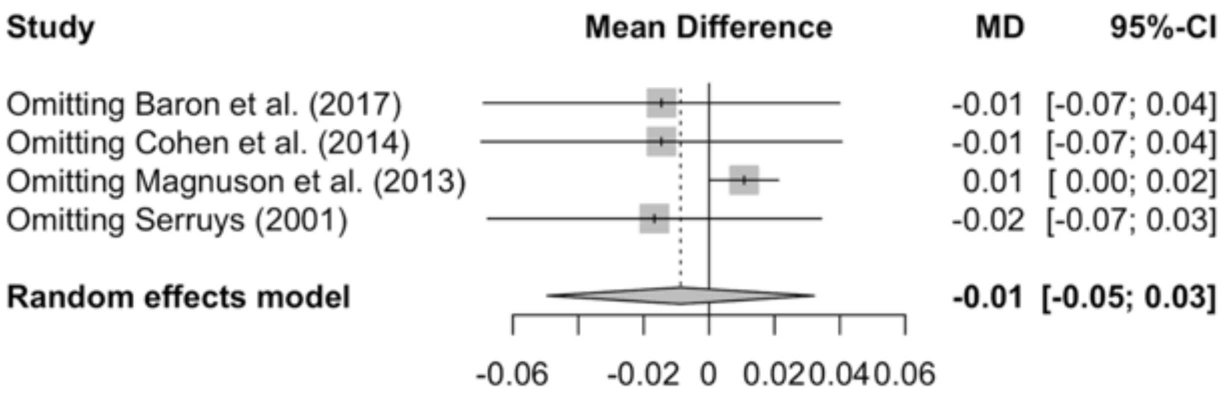

CI, confidence interval; MD, mean difference.

Figure S12. Funnel plot for Euro-QoL-5D (EQ-5D)

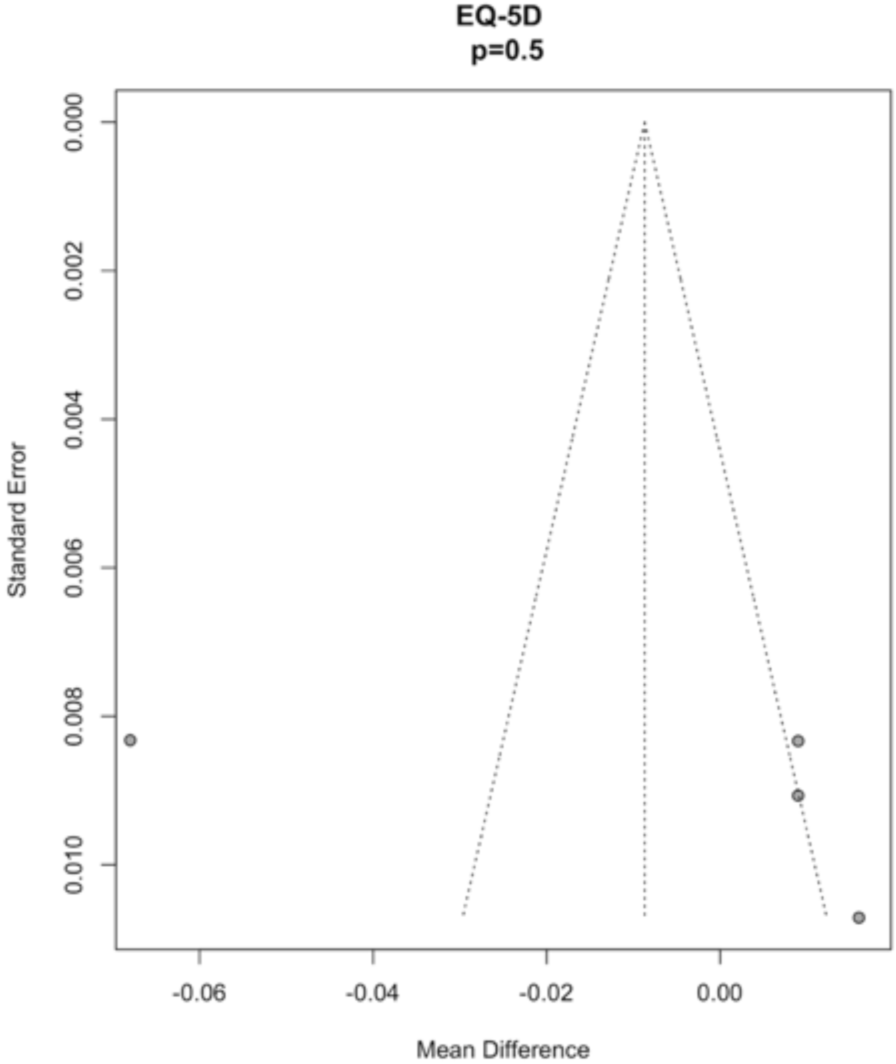

Figure S13. Mean gains in Short Form- Physical Component (SF-PC) during the follow-up.

CABG, coronary artery bypass grafting; EXCEL, Evaluation of XIENCE versus Coronary Artery

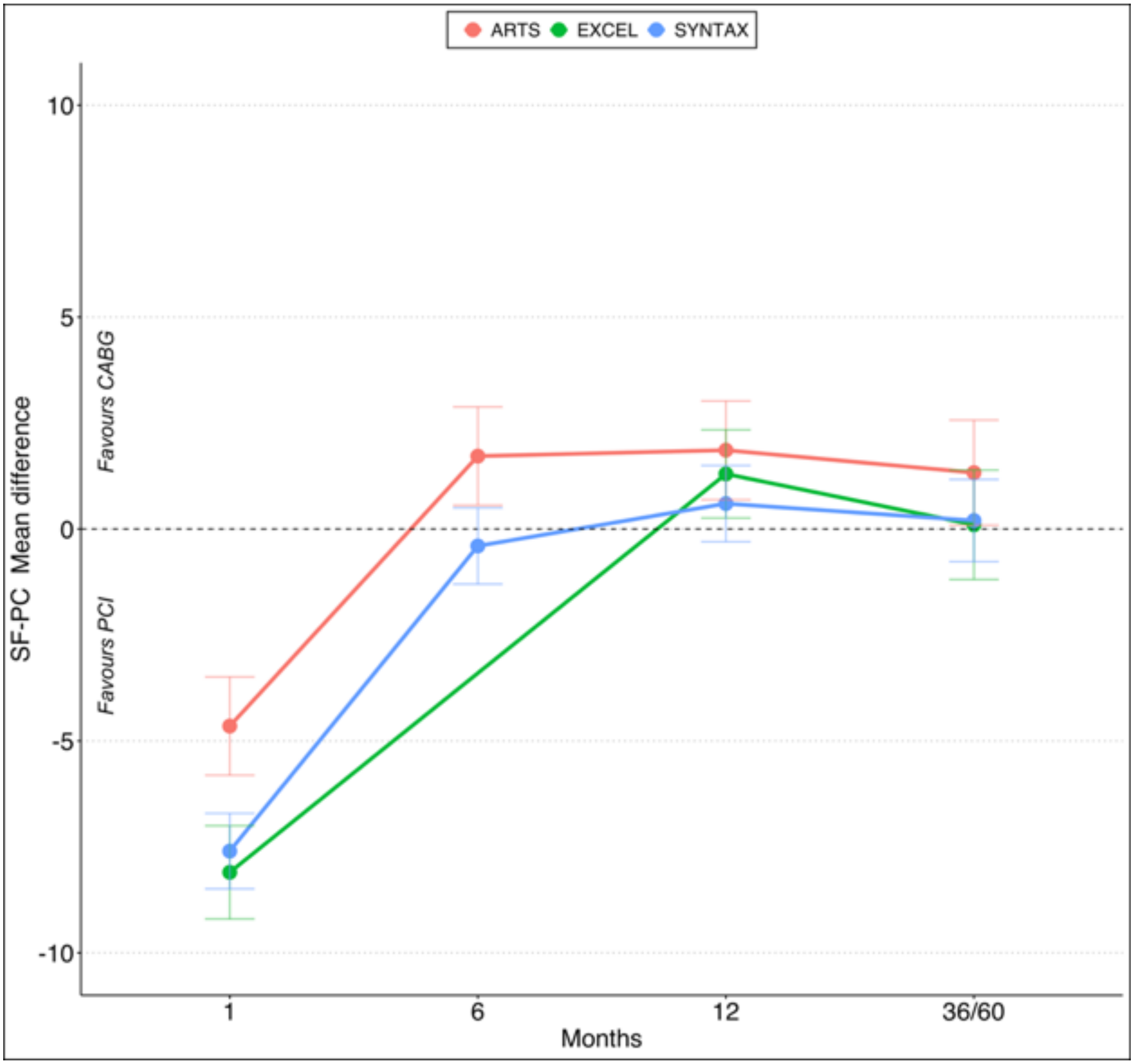

Bypass Surgery for Effectiveness of Left Main Revascularization; FREEDOM, Future Revascularization Evaluation in Patients with Diabetes Mellitus: Optimal Management of Multivessel Disease; PCI, percutaneous coronary intervention; SF-PC, Short Form-Physical Component; SoS, Stent or Surgery; SYNTAX, Synergy between PCI with TAXUS and Cardiac Surgery.

Figure S14. LOO analysis: SF-12 (PCS)

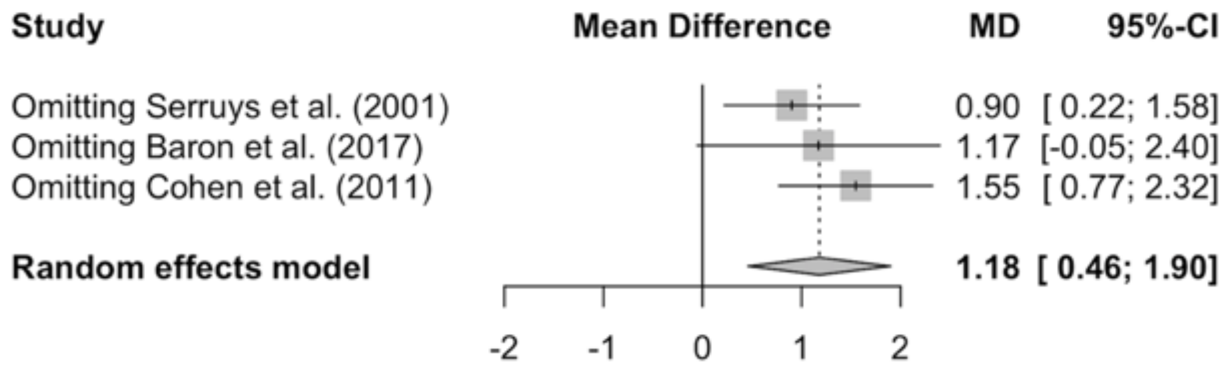

CI, confidence interval; MD, mean difference.

Figure S15. Funnel plot for the Short Form-Physical Component (SF-PCS)

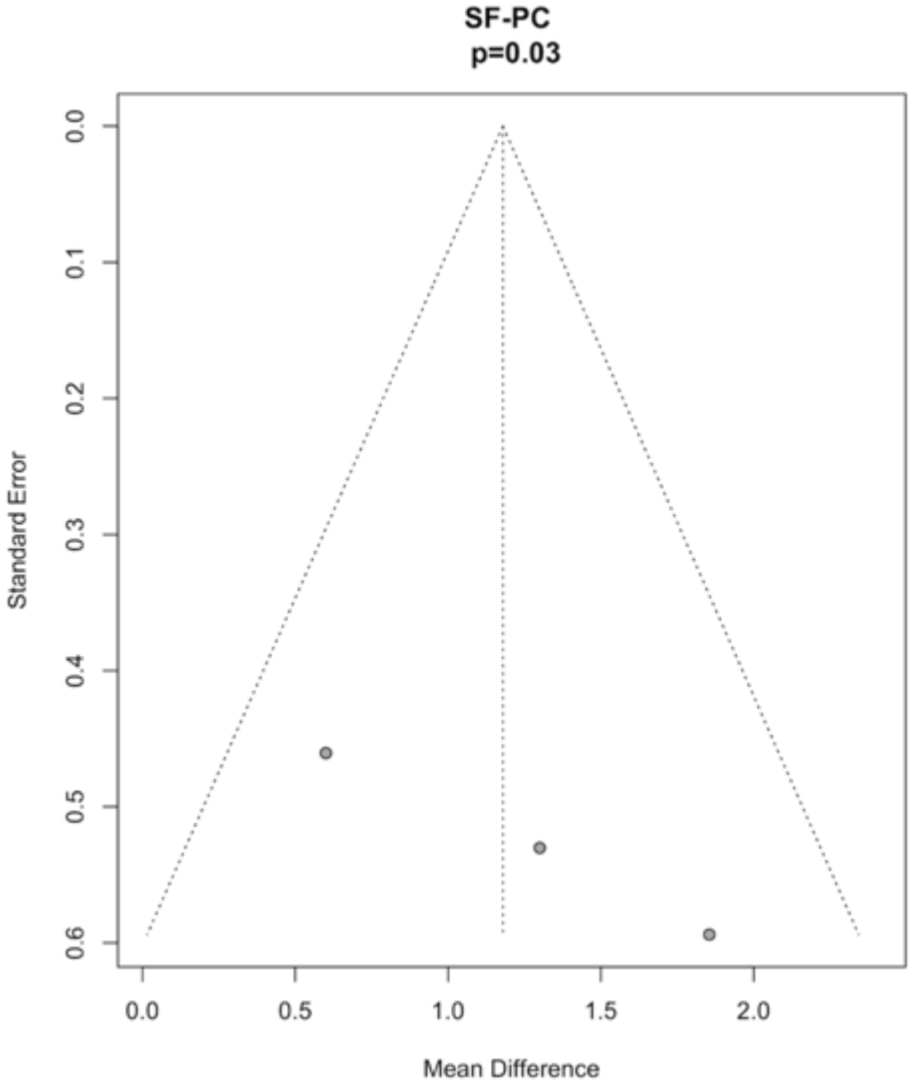

Figure S16. Mean gains in Short Form-Mental Component (SF-MC) during the follow-up.

CABG, coronary artery bypass grafting; EXCEL, Evaluation of XIENCE versus Coronary Artery

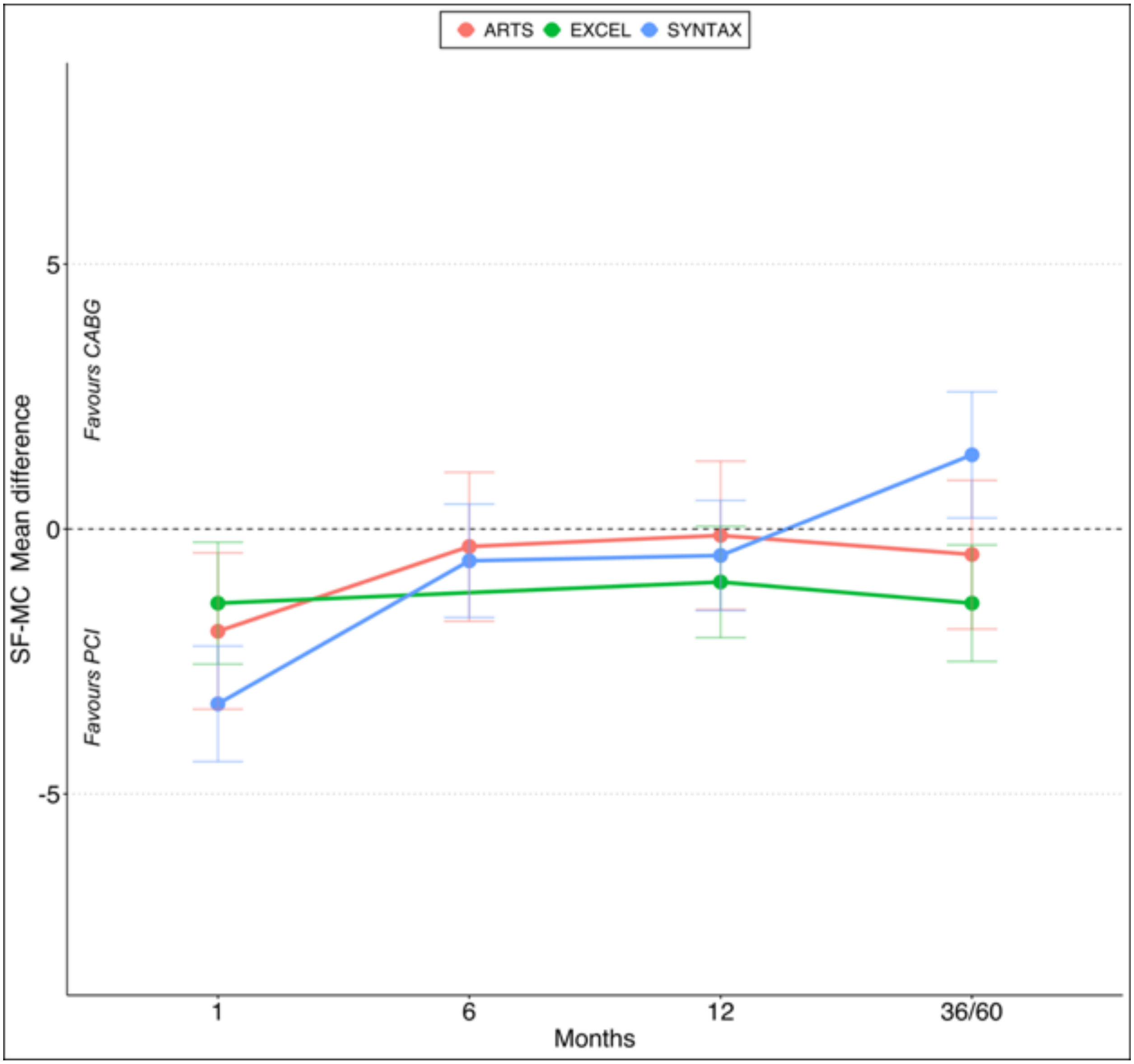

Bypass Surgery for Effectiveness of Left Main Revascularization; FREEDOM, Future Revascularization Evaluation in Patients with Diabetes Mellitus: Optimal Management of Multivessel Disease; PCI, percutaneous coronary intervention; SF-MC, Short Form-Mental Component; SoS, Stent or Surgery; SYNTAX, Synergy between PCI with TAXUS and Cardiac Surgery.

Figure S17. Leave-one-out analysis for the Short Form-Mental Component

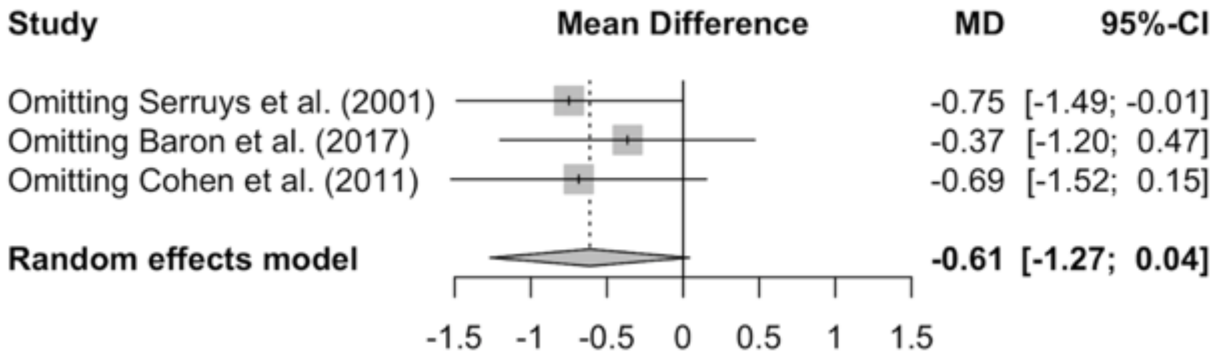

CI, confidence interval; MD, mean difference.

Figure S18. Funnel plot for the Short Form-Mental Component (SF-MC)

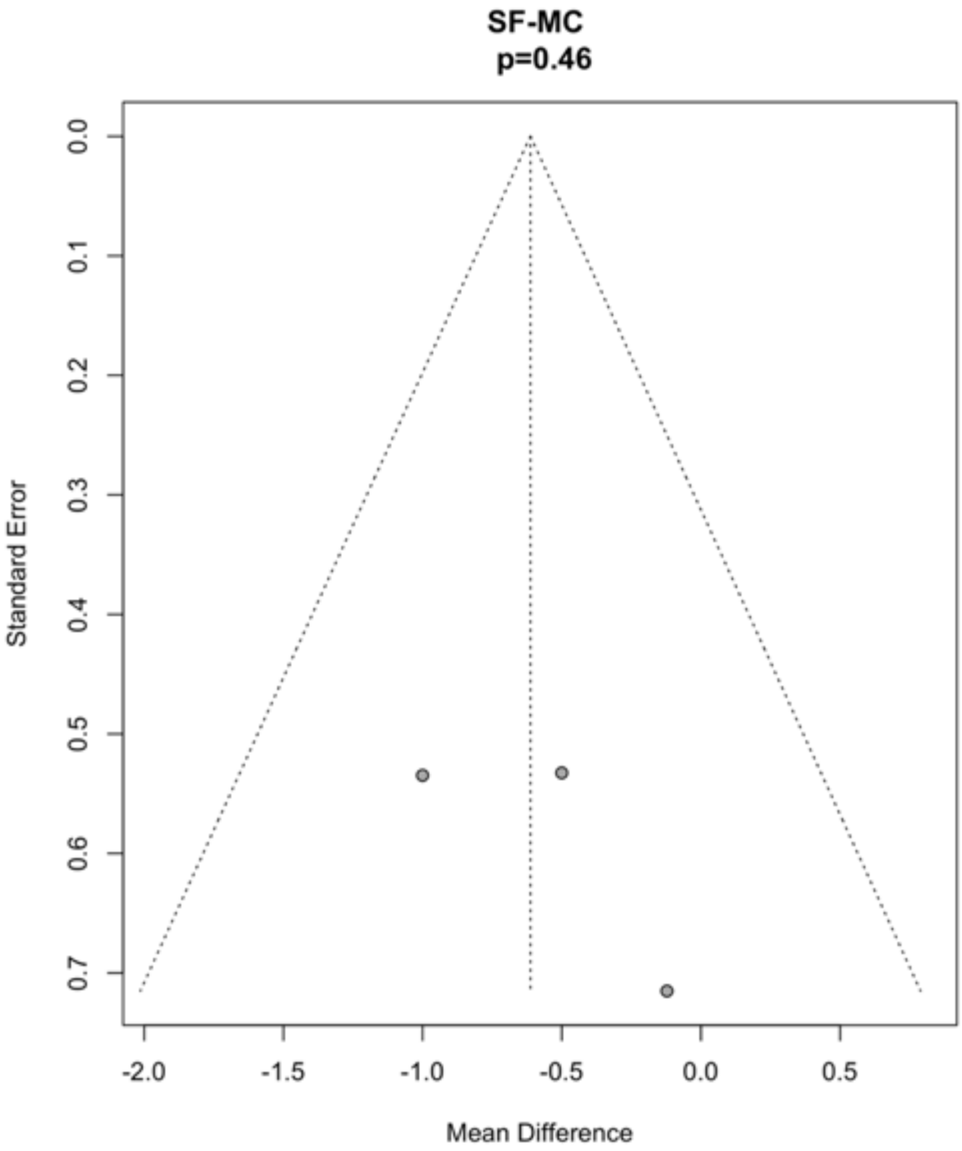

Supplement: Supplementary file 1 — Table S1 Figures S1–S18 [file JAH3-12-e030069-s001.pdf]
